# Daunomycin Nanocarriers with High Therapeutic Payload for the Treatment of Childhood Leukemia

**DOI:** 10.3390/pharmaceutics17091236

**Published:** 2025-09-22

**Authors:** Rosa M. Giráldez-Pérez, Elia M. Grueso, Antonio J. Montero-Hidalgo, Cristina Muriana-Fernández, Edyta Kuliszewska, Raúl M. Luque, Rafael Prado-Gotor

**Affiliations:** 1 Department of Cell Biology, Physiology and Immunology, Faculty of Sciences, University of Cordoba, 14014 Cordoba, Spain; 2 Department of Physical Chemistry, Faculty of Chemistry, University of Seville, 41012 Seville, Spain; 3 Maimonides Biomedical Research Institute of Cordoba (IMIBIC), Reina Sofia University Hospital (HURS), 14004 Cordoba, Spain; 4 Chemtra, 47-300 Krapkowize, Poland; 5 CIBER Physiopathology of Obesity and Nutrition (CIBERobn), 14004 Cordoba, Spain

**Keywords:** chemotherapy, gold nanoparticles, nanocarriers, gemini surfactants, daunomycin, childhood leukemia

## Abstract

**Background/Objectives**: Malignant neoplasms in children include leukemias. The main types are B-cell acute lymphoblastic leukemia (B-ALL) and acute myeloid leukemia (AML). Treatments are expensive, which is a particular problem in low-income countries. The main objective of this work was to develop specific nanosystems with small amounts of drug, allowing for affordable treatments. To this end, we designed ternary gold nanosystems (Au@16-Ph-16/DNA–Dauno) composed of daunomycin, a DNA biopolymer as a stabilizer, and the cationic surfactant gemini (TG) as a compacting agent for the DNA–daunomycin complex. **Methods**: Fluorescence, UV–visible, and CD spectroscopy, DLS and zeta potential, cell viability assays, TEM, AFM, and confocal microscopy were used to characterize and optimize nanocomposites. **Results**: The nanoparticles (Au@TG) obtained were small, stable, and highly charged in solution, allowing for optimal absorption and efficacy, capable of inducing the aggregation of the ternary nanosystem upon entering the cell, further enhancing its anticancer effect. Using nanoparticles, treatments can be redirected to the site of action, increasing the solubility and stability of the drug, minimizing the side effects of traditional treatments, and helping to overcome resistance to chemotherapy **Conclusions**: A significant decrease in the growth of pediatric B-ALL-derived cell lines (SEM and SUP-B15), constituting a potential and more affordable therapy for this type of pathology.

## 1. Introduction

According to the World Health Organization, there are approximately 400,000 children in high-income countries, with access to comprehensive care services. Of all the above-mentioned children only 80% of them affected by cancer are cured, but in low- and middle-income countries, less than 30% are cured [1,2,3,4,5,6]. Acute myeloid leukemia (AML) is a rapidly progressing myeloid neoplasm characterized by the clonal expansion of immature cells derived from the bone marrow. These cells, called blasts, circulate in the peripheral blood and bone marrow. This expansion leads to ineffective erythropoiesis and megakaryopoiesis, resulting in bone marrow failure, which manifests clinically as relatively rapid bone marrow failure compared to chronic leukemias. Patients present with rapid cytopenias, decreased white blood cells, hemoglobin, or platelets, circulating blast cells in peripheral blood, easy bruising or bleeding, or recurrent infections. Other symptoms include renal failure caused by autologous tumor lysis syndrome [7,8,9,10,11]. Acute lymphocytic leukemia (ALL) is a malignant neoplasm of B or T lymphoblasts characterized by the uncontrolled proliferation of abnormal and immature lymphocytes and their progenitors, which ultimately leads to the replacement of bone marrow elements and other lymphoid organs. It can cause anemia, thrombocytopenia, and neutropenia due to the replacement of bone marrow by the tumor. In addition to other symptoms such as fatigue, easy or spontaneous bruising or bleeding, infections, hepatomegaly, splenomegaly, and lymphadenopathy may be observed, and it can often affect the central nervous system [12,13,14,15,16]. Common childhood malignancies include leukemias, accounting for almost 40%, with the main types being B-cell acute lymphoblastic leukemia (B-ALL) and acute myeloid leukemia (AML) [17].

Nanomedicine, and specifically nano-oncology integrated with personalized medicine, is gaining a lot of attention due to the great potential of nanoparticle-based drugs for treating cancer. The use of nanoparticles can significantly improve the administration of anticancer treatments. This is because nanoparticles can alter the solubility of drugs and increase their stability within the body. In addition, they can be designed to specifically target particular cells or tissues, minimizing side effects compared to traditional treatments. By leveraging the unique properties of nanoparticles, such as their ability to deliver drugs with precision, reduce systemic toxicity, and improve therapeutic efficacy, nanotechnology has the potential to revolutionize cancer diagnosis and treatment. Drug-conjugated nanocarriers can help improve the doses of drugs administered in the body and increase their circulation in the bloodstream [18,19].

In this regard, nanoparticles are designed to modify pharmacokinetics and thus improve drug activity, acting as precise carriers for targeted delivery to specific cells or tissues on which they must act, thereby maximizing the therapeutic index of the drug [18,19]. In this scenario, gold nanoparticles stand out for their biocompatibility, low toxicity, and unique optical properties, making them ideal for medical applications, especially when synthesized using methods that ensure uniformity in size and shape, facilitating their entry into cancer cells [20,21].

This work aims to find nanosystems where a small amount of anticancer drug is needed, to allow more affordable treatment. In addition, attempting to transport these nanosystems directly to the areas where the drug should act would minimize the side effects that often occur in treated children. Gold ternary nanosystems are formed with anticancer drugs, such as daunomycin (Dauno) [22,23,24], frequently used to treat various types of cancer such as leukemia [25,26,27,28,29,30]. For example, Vyxeos^®^ (CPX-351) [31] is a liposomal formulation of a fixed combination of daunorubicin and cytarabine in a 1:5 molar ratio, which is the ratio that has demonstrated maximum antitumor synergy in vitro and in vivo. It was designated as an orphan drug by the European Medicines Agency–European Union (EMA) in 2012 for the treatment of AML and authorized by the EMA in 2018. CPX-351 is indicated as monotherapy, especially in adult patients diagnosed with high-risk AML [32]. They have a high concentration of daunorubicin. There is insufficient data in the pediatric population or in patients over 85 years of age. The drug is safe in cases of hepatic insufficiency with bilirubin below 50 μM (2.9 mg/dl), but there is no data in cases of more severe hepatic insufficiency. No significant difference in clearance was observed in patients with mild or moderate renal impairment (creatinine clearance > 30 mL/min), and the potential effects in patients are unknown. Therefore, the design of effective nanocarriers in children using only small amounts of Dauno is a promising therapy for pediatric patients with leukemia and severe renal impairment [33]. Besides gold and Dauno anticancer drug, we used a cationic surfactant of the geminis type (TGs) and a DNA biopolymer [20,21]. In this case, we used the cationic surfactant 16-Ph-16. As a result, the low size and high zeta potential values of the obtained nanosystem confer great drug solubility and stability in solution, which facilitates its optimal absorption and effectiveness [20,34]. The use of calf thymus DNA (DNA) is remarkable, constituting a biomimetic and non-toxic linking material that will help to facilitate the entry of the nanosystem into the cells [20,21]. Thus, a condensed highly charged biocompatible nanosystem with high colloidal stability as supported by UV–visible and CD spectroscopies, TEM and AFM, DLS, and zeta potential techniques. Once the compacted Au@16-Ph-16/DNA–Dauno nanosystems were characterized and transferred through the cell, studies of toxicity and internalization of them were performed using cell viability analysis, TEM, and confocal microscopies. For this purpose, tumor cell lines from specific leukemias in children such as SUP-B15 [35,36] were used. SUP-B15 is a B lymphoblast cell line. It was first isolated from an 8-year-old male patient with acute lymphoblastic leukemia (B-ALL). It is a line normally used for research. On the other hand, the SEM cell line, specifically for girls, was isolated from the peripheral blood of a 5-year-old girl with acute lymphoblastic leukemia (B-ALL) [37] and was used as a possible therapy, that was more affordable and easier to prepare, against these types of cancers. In conclusion, the goal is to develop a daunomycin nanocarrier that could contribute significantly to the efficacy and treatment of childhood leukemia. To this end, the efficacy of gold nanosystems combined with the drug daunomycin, DNA, and the gemini cationic surfactant will be evaluated.

## 2. Materials and Methods

### 2.1. Materials

The products used are sodium borohydride (NaBH_4_) (Lancaster, PA, USA). DNA, daunomycin (Dauno), hydrogen tetrachloride (III) trihydrate, sodium cacodylate, and 3-aminopropyltriethoxysilane (APTES) were purchased from Sigma-Aldrich–Merck KGaA (Darmstadt, GER). To verify the existence of possible protein contamination, the absorbance ratio of the DNA solution at 260 and 280 nm was checked [38]. As a result, a ratio A_260 nm_/A_280 nm_ = 1.87 was obtained, which indicates no contamination. The average number of base pairs per DNA molecule, determined by agarose gel electrophoresis with ethidium bromide, was greater than 10,000 bp. DNA concentrations in base pairs (C_DNA_) were determined spectrophotometrically as double-stranded DNA (ds-DNA) concentrations at 260 nm based on a molar absorbance of DNA of 13,200 M^−1^ − cm^−1^ [39].

#### 2.1.1. Synthesis of N,N’-[1,3-Phenylenebis(methylene))bis[N,N’-Dimethyl-N-(1-Hexadecyl)]-Ammonium Dibromide, 16-Ph-16

For the synthesis of the gemini surfactant 16-Ph-16, α,α’-dichloro-p-xylene (11.9 g and 0.068 mmol) was mixed with dry acetonitrile and a solution of N, N-dimethylhexadecylamine (40.1 g and 0.15 mmol) in 150 mL of acetonitrile. The sample was refluxed during 96 h at 355 K for the removal of the solvent and it was then recrystallized from a mixture (4/1) of acetone/hexane. A white solid was obtained upon cooling with a yield of 86%. The CMC of the 16-Ph-16 surfactant gave a value of (8.0 ± 0.4) µM [40]. Characterization is shown in Figure S1.

#### 2.1.2. Synthesis of Au@16-Ph-16 Precursor Gold Nanoparticles

To obtain the functionalized Au@16-Ph-16, 30 mL of 16-Ph-16 µM was mixed with 390 μL of 23 mM HAuCl_4_. After 5 min of stirring in the dark, 100 μL of a 0.4 M NaBH_4_ aqueous solution was added, keeping the mixture stirring and in the dark for 15 min, acquiring a reddish hue in the dark [20]. The ratio between C_16-Ph-16_ and the critical micelle concentration (CMC) was 5, since a previous study established that when this ratio was between 1 and 20, stable and monodisperse gold nanoparticles coated with the surfactant were obtained in aqueous solution [20]. With an Au@16-Ph-16 concentration of 56 nM, three nanosystems (abbreviated as N_I_) designated as N_1_, N_2_, and N_3_ were studied (see Table 1).

#### 2.1.3. Synthesis of Au@16-Ph-16/DNA–Dauno Gold Nanosystem

Once the synthesis of the precursor Au@16-Ph-16 was achieved, the Dauno transporter, Au@16-Ph-16/DNA–Dauno, was synthesized starting from DNA/Dauno complexes prepared under saturation, X = C_Dauno_/C_DNA_ = 0.10 according to the previously estimated binding constant for the daunomycin–calf thymus DNA interaction, K_DNA/Dauno_ = (7.0 ± 0.25) X 10^5^ M [41]. Method tuning also required the prior selection of the optimal relative Au@16-Ph-16/DNA concentrations, R = C_Au@16-Ph-16_/C_DNA_ for obtaining compacted and smaller Dauno nanocarriers for cell internalization. According to the results from CD, DLS, and AFM techniques (see Section 3.2 for more details), this R ratio was stablished in 2.2 × 10^−5^, since below this ratio, the amount of Au@16-Ph-16 nanoparticles added to the DNA–Dauno complex was not sufficient to completely compact the nanosystem, and above this ratio, self-aggregation processes between the Au@16-Ph-16/DNA–Dauno nanosystems began to become evident. Three Au@16-Ph-16/DNA–Dauno nanosystems (C_I_ in abbreviated form) designated as C_1_, C_2_, and C_3_ were prepared, varying Dauno–drug concentration and thus, reactant concentrations in ternary system (see Table 1)

#### 2.1.4. Cell Lines and Culture Conditions

Cell lines derived from pediatric B-ALL (SEM and SUP-B15) were obtained from the Leibniz Institute DSMZ (#ACC 546 and #ACC 389, respectively) and cultured according to the supplier’s recommendations. Specifically, SEM was cultured with IMDM media (12440053; ThermoFisher Scientific. Waltham, MA, USA), supplemented with 10% FBS (10270106; ThermoFisher Scientific. Waltham, MA, USA) and glutamine (2 mM; X0550; Biowest), while SUP-B15 cells were cultured with McCoy’s 5A (26600023; ThermoFisher Scientific. Waltham, MA, USA), supplemented with 20% FBS and glutamine (2 mM). All cell lines were maintained in a humidified incubator with 5% CO_2_ at 37 °C. Cell line identity was validated by short tandem repeats sequences (STRs) analysis. All cell lines were tested for mycoplasma contamination by PCR, as previously reported [42].

### 2.2. Methods

#### 2.2.1. Cell Viability Assays

Cell viability was examined by Resazurin assay (CA035, Canvax Biotech. Valladolid, SP) following manufacturer’s instructions. Briefly, cells were plated in 96-well plates at a density of 5000 cells/well. Cells were treated with different N_I_, C_I_, N_I_ + C_I_ formulations and the corresponding free Dauno_I_ concentrations as previously stated (see Table 1). Cell viability was then evaluated at 24 and 48 h after incubation. Fluorescence (I_560 nm_) was evaluated using the VANTAstar (BMG Labtech, Ortenberg, GER) after 3 h of incubation with resazurin reagent at 10%. Moreover, the fluorescence of the different N_I_, C_I_, N_I_ + C_I_ and Dauno_I_ compounds of each treatment was evaluated in the absence of cells at the same experimental condition (λ_emission_ = 560 nm) to consider the fluorescence contribution of each individual compound by itself. Thus, the corrected fluorescence at I_560 nm_ value was obtained as in a previous paper [21] and considering baseline fluorescence of the medium itself and of each specific system by normalizing each experimental condition by its corresponding baseline fluorescence in a cell-free well.

All in vitro experiments were performed at least 3 different times (*n* ≥ 3 biological replicates), and with at least 2 technical replicates unless otherwise indicated. Standard error of the mean was calculated and represented on the same graph. Mean of technical replicates per biological replicate was used for hypothetical testing. Statistical differences were calculated using one-way ANOVA analysis corrected by multiple comparisons with Dunnet testing. Statistical significance was considered when *p* < 0.05. All analyses were assessed using GraphPad Prism 8 (GraphPad Software, Boston, MA, USA).

#### 2.2.2. UV/Vis Spectroscopy

The absorbance of the samples was measured using a Zuzi double-beam spectrophotometer (model 4260/50, Valencia, Spain) with a wavelength of ±0.3 nm and a spectral bandwidth of ±0.5 nm. The stability of the Au@16-Ph-16 nanoparticles was monitored with UV-Vis spectra in a range from 200 to 800 nm and over a period of one month.

#### 2.2.3. Fluorescence Spectroscopy

The fluorescence of the different compounds was measured at 298.0 K using a Hitachi F-2500 spectrofluorometer (Hitachi High Technologies America, Inc., Schaumburg, IL USA) at a speed of 1500 nm/min with an excitation and emission slit of 5.0 nm and a PMT voltage of 700 V. The concentrations of C_Dauno_ were set at 10 μM, C_DNA_ at 100 μM, and the precursor C_Au@16-Ph-16_ ranged from 0.187 to 51.3 nM. The wavelengths were set at 480 nm for the excitation and 555 nm and 592 nm for the emission.

#### 2.2.4. Circular Dichroism (CD) Spectroscopy

Circular Dichroism (CD) spectra used a BioLogic Mos-450 spectropolarimeter (Barcelona, SP), using a standard quartz cell with a 10 mm pitch length. Spectra were expressed in terms of molar ellipticity (θ). Sweeps were taken from 210 to 340 nm and averaged from 5 to 10 runs at a constant temperature of 298.0 K with a 10 min equilibration before each sweep. All the spectra were recorded at a fixed concentration of double-stranded DNA, C_DNA_ = 100 μM, and Daunomycin, C_Dauno_ = 10 μM. The Au@16-Ph-16 concentrations, C_Au@16-Ph-16_, were 0, 0.0186, 0.0373, 0.0747, 0.112, 0.149, 0.187, 0.224, 0.280, 0.373, 0.560, 0.747, 1.12, 1.49, 1.87, 2.24, 2.61, 4.11, 4.85, 5.12, and 5.18 nM.

#### 2.2.5. Atomic Force Microscopy Experiments

The resonance at which the atomic force microscopy images were obtained was 40 kHz and an elastic constant of 42 N/m, using silicon cantilevers (Pointprobe model, Nanoworld, Neuchâtel, Switzerland). The microscope used was a Molecular Imaging Picoscan 2500 (Agilent Technologies, Madrid, Spain). The images were acquired at 256 × 256 dpi and then flattened to avoid any background slope [43]. To modify the mica, it was incubated with 0.1% (*v*/*v*) APTES.

#### 2.2.6. Dynamic Light Scattering (DLS), Polydispersity Index (PDI), and Zeta Potential Measurements

The size and distribution of the nanoparticles were characterized using a Malvern Zetasizer Model ZS-90 (Malvern Panalytical Ltd., Worcestershire, UK). Multiple measurements were taken to obtain the average hydrodynamic diameter and zeta potential value using a laser Doppler velocimeter, in formulations under different conditions of both ionic strength and distilled water. Zeta potential was measured from electrophoretic mobility placing the nanoparticles into an electric field. Then, using the Smoluchowski equation and assuming a spherical form for the particles, the zeta potential was calculated [44]. Previous control experiments were measured in PBS buffer (0.01×, ionic strength = 1.63 mM, and pH = 7.4) and in the absence and in the presence of FBS, establishing that pure water is the better dispersing solvent for the nanocarriers. Moreover, the release kinetic of C_3_ nanocomplex in PBS with and without FBS was measured, evaluating the decrease in the mean size of the nanocomplex over time. This process, which takes approximately 30 min in PBS, is much faster in the presence of FBS. At least five size measurements were taken for each sample, and the relative error of the average hydrodynamic diameter was calculated to be <5%. On the other hand, six zeta potential (ζ) measurements were taken, measuring the electrophoretic mobility of the particles in the samples, with a relative error of <5%. Polydispersity index (PDI), an important index that evaluates the spread of nanoparticle size distribution, was calculated, considering the squared of the standard deviation divided by the squared of the mean particle diameter (PDI = (σ/d)^2^) [45].

#### 2.2.7. TEM and EDS Measurements

Energy Dispersive Spectroscopy (EDS) analysis of the Au@16-Ph-16 nanoparticles was performed using a high-resolution transmission electron microscope (200 kV) TALOS F200S (FEI company, Hillsboro, OR, USA), which demonstrated the presence of gold. In addition, microphotographs of the nanoparticles were taken, and the size of approximately 350 nanoparticles was studied using the ImageJ 1.52a software (2018, Bethesda, MD, USA). The results are given in Figure S2 which provides a mean diameter of (3.6 ± 0.9) nm for the core size of precursor Au@16-Ph-16 gold nanoparticles.

On the other hand, to verify the presence of gold inside the cells, a Zeiss EVO LS15 microscope was used, which allows EDS analysis to be performed inside the cells without affecting organic tissues, since the power of the TALOS does not allow such analysis in organic tissue. In this case, the presence of gold inside cells treated with Au@16-Ph-16, Au@16-Ph-16/DNA–Dauno, and Au@16-Ph-16/DNA–Dauno + Au@16-Ph-16 is shown.

To verify the internalization of nanoparticles and nanosystems into cells, the samples were placed in an automatic sample processor, and finally the samples were treated with a 1% osmium tetroxide solution, followed by a 2% uranyl acetate solution, then dehydrated and successively incorporated into epoxy resin. Samples were then cut into thin sections to determine the best ones for study. To do this, the samples were stained with toluidine blue and observed under an optical microscope. Ultrathin sections of 70 nm were then cut with a diamond-edged blade, deposited on copper grids, and observed with a Zeiss Libra 120 microscope (Zeiss, Jena, GER) to observe both the nanoparticles and nanosystems and their internalization into cells. (For more details, see Point 2 of the Supplementary Material).

#### 2.2.8. Confocal Microscope

The presence of nanoparticles and nanosystems was analyzed inside the cell using a confocal microscope (LSM7DUO, Carl Zeiss Microscopy, Jena, GER) and ZEN 2011 software. For better visualization, cell nuclei were stained with Fluoroshield™ with DAPI (F6057, Sigma-Aldrich–Merck KGaA, Darmstadt, GER) (For more details, see Point 3 of the Supplementary Material). To avoid autofluorescence in microscopy, the excitation light intensity was reduced to minimize the irradiation that causes autofluorescence and fluorescence fading. Controls were performed by inspecting the cells without any type of label so that no labeling was observed in any laser except for the cell nuclei, which is illuminated by the fluorescent dye 4′,6-diamidino-2-phenylindole (DAPI), specific for DNA found in the nuclei, thus demonstrating that there is no intrinsic fluorescence belonging to the cells. The cell edge was monitored with bright field micrography, and nanoparticles and nanosystems were labeled with green fluorescence; no fluorescence was found for other lasers.

## 3. Results and Discussion

A ternary nanosystem (Au@16-Ph-16/DNA–Dauno) has been designed, consisting of daunomycin (Dauno), frequently used to treat several types of cancer such as leukemia, with the DNA biopolymer as a stabilizer and the cationic surfactant gemini 16-Ph-16 as a compacting agent of the DNA–Dauno complex. The result is small, highly stable nanoparticles with a high charge in solution which facilitates their optimal uptake and effectiveness. However, the main outcome of this work is the fact that the bare nanoparticle itself (Au@16-Ph-16) at the appropriate concentration can induce the aggregation of the ternary nanosystem once it has entered the cell, further enhancing their anticancer effect. A significant decrease in the growth of tumor cells of specific leukemias was registered for boys (SUP-B15, cell line) and girls (SEM, cell line), constituting a possible therapy, that was more affordable and easier to prepare, against these types of cancers.

### 3.1. Studies of the Charge, Size and Stability of Au@16-Ph-16 and Au@16-Ph-16/DNA–Dauno Nanosystems

Au@16-Ph-16 particles were found to have a diameter of 3.6 ± 0.9 nm (Figure S2). The stability of this nanosystem was performed from synthesis up to one month (see Figure S3) at a fixed temperature of 298 K. As is well known, both the temperature and the type of medium used as a dispersant for the particles can significantly affect their stability. In particular, the increase in temperature decreases the viscosity and density of the dispersing medium, leading to faster particle settling and reducing their stability [46].

Of the concentration series performed, the most suitable are three, which are named as N_1_, N_2_ and N_3_ for AuNPs (Au@16-Ph-16) (Table 2 and Figures S4 and S5) and C_1_, C_2_ and C_3_ for nanosystems (Au@16-Ph-16/DNA–Dauno) (Table 3 and Figures S6 and S7). Note that the different samples were measured in an aqueous medium, as control experiments highlighted the instability of the samples in serum (see Table S1).

The zeta potential of a nanoparticle represents the charge of the nanoparticle, around the value of ±30 mV, which is the optimal value to have the highest nanoparticle stability. As can be seen in Table 2 and Table 3, the values of our samples are greater than ±30 mV, thus ensuring their optimum stability. As expected, the charge of the gold nanoparticle stabilized with the cationic gemini surfactant 16-Ph-16 is highly positive. However, the binding of the biopolymer to the Au@16-Ph-16 precursor causes a charge reversal in the nanoparticle, which becomes highly negative. This is consistent with the highly favored binding of the large size of the biomolecule to the precursor. In such a way that the conjugation of the positively charged nanoparticle with the large size DNA containing a high number of negatively charged phosphate groups generates a nanocomplex with a high overall negative charge in solution.

Regarding the results of the mean size distribution of the precursor and the nanocarriers, it should first be noted that the size measured by DLS for the precursor is slightly larger than that measured by TEM. This is because the TEM microscopy technique only allows the diameter of the gold center to be measured, while the DLS technique measures the contribution of the stabilizing agent, in this case the micelles of the 16-Ph-16 surfactant, which becomes more important as the HAuCl_4_:16-Ph-16 ratio used for the synthesis decreases [47,48]. On the other hand, the binding of the DNA–Dauno complex to the precursor generates small-sized nanocomplexes (see Table 3), considering the average size of calf thymus DNA fibers in aqueous solution, which ranges around 800 nm [49]. This is indicative of a possible compaction of the biomolecule induced jointly by the nanoparticle and the anticancer agent.

Finally, the polydispersity index (PDI) is an important parameter and criterion to consider the uniformity of the size distribution of a nanoparticle or polymer nanosystem. When a low PDI value is obtained close to 0 (PDI < 0.1), the nanosystem can be considered as being monodisperse and uniform, with a consistent size distribution. However, a high PDI value (PDI > 0.1) is indicative of a broad and heterogeneous size distribution for the particles [45,50]. Table 3 and Table 4 show the obtained values of PDI for the Au@16-Ph-16 precursor nanoparticles and the compacted nanocarriers. As all PDI values obtained are close to zero and in any case below the threshold value of 0.1, it can be deduced that the different nanosystems are monodisperse and have a uniform size distribution. All of this indicates high stability in the solution of the different formulations, which helps to guarantee their potential therapeutic application and optimize the transport of the drug daunomycin in its early stages of internalization.

### 3.2. Fluorescence Spectroscopy: DNA/Dauno and Au@16-Ph-16/DNA–Dauno Binding Studies

**Figure 1:**
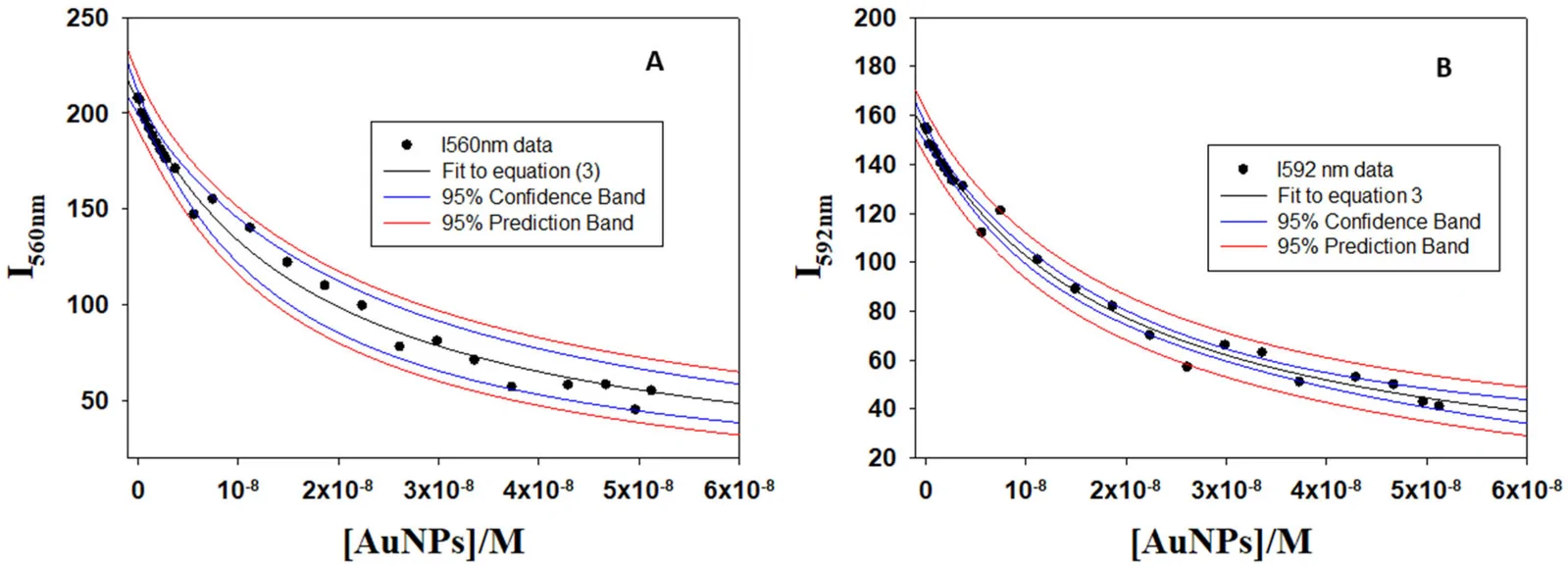
Plot of the fluorescence intensity experimental data versus AuNPs concentrations at T = 25.0 °C. Symbols (●) are experimental data and black line is the best fit using Equation (3); 95% confidence and prediction (residuals) bands are also displayed as lines in blue and red, respectively. (**A**) fluorescence intensity fixed at 555 nm, (**B**) fluorescence intensity fixed at 592 nm.

**Scheme 1:**
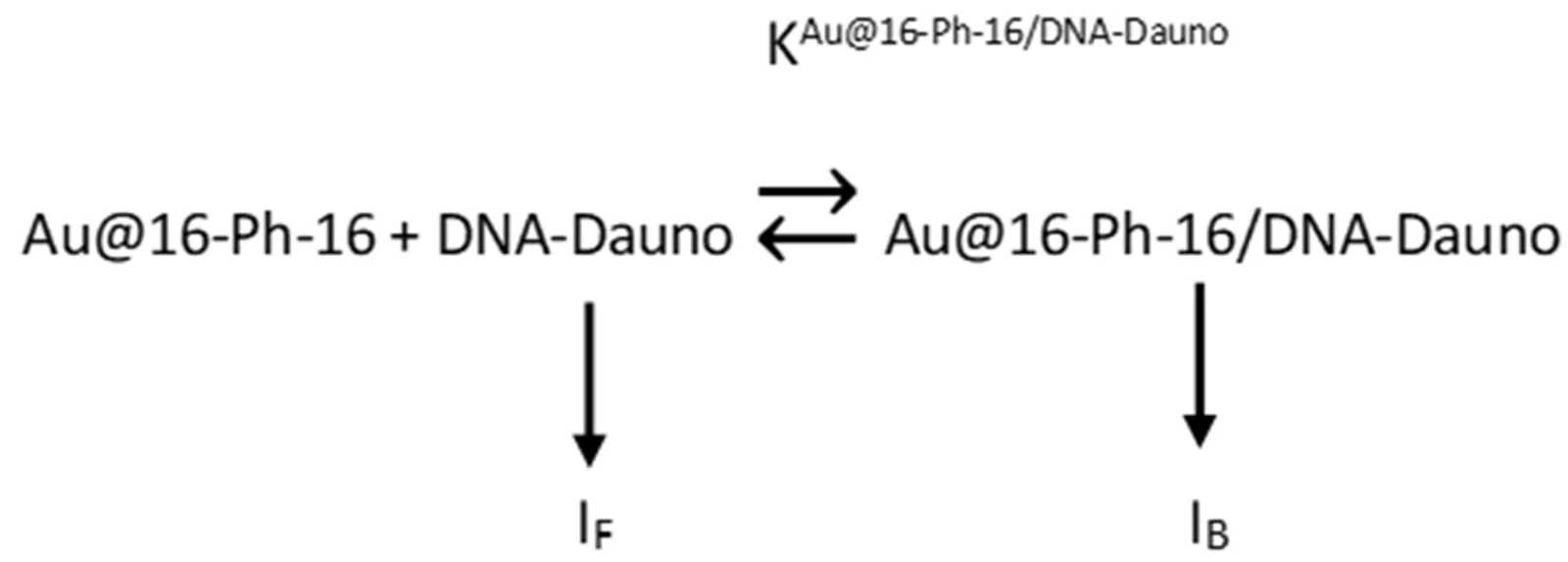
Au@16-Ph-16/DNA–Dauno interaction.

The binding of daunomycin to DNA is highly favorable from an energy standpoint; with an equilibrium constant of K = (6.7 ± 0.9) × 10^5^ (M^−1^) in water at 298 K, the fluorescence drug binds to DNA by the process of intercalation. Moreover, the thermodynamic profile shows that hydrogen bond interactions are responsible for the high stability of the DNA–Dauno complex [51]. Evaluating the equilibrium constant of the nanocomplex formation that will transport daunomycin at the cellular level is essential to ensure the stability and integrity of the synthesized nanosystem. In this sense, fluorescence spectroscopy is a versatile and important method for quantifying the interactions of small molecules with DNA. As is well known, the fluorescence emission spectrum of daunomycin has two emission maxima at 555 and 592 nm. The intensity of these maxima decreases because of interaction with DNA, with no evidence of changes in the position of these maxima [52]. Figure 1A-B shows the changes in the fluorescence intensity of the Au@16-Ph-16/DNA–Dauno system at 555 and 592 nm as increasing amounts of Au@16-Ph-16 nanoparticles were added to a solution containing the DNA–Dauno complex. The experiments were carried out at a fixed biomolecule and drug concentration and under saturation condition. Based on these changes in fluorescence of DNA-D complex at a fixed wavelength, it is possible to quantify the equilibrium constant of the Au@16-Ph-16/DNA–Dauno interaction using the Pseudophase Model [53]. According to this model, the reaction between the gold nanoparticles and DNA–Dauno complex can take place in both the aqueous and the DNA pseudophases with two different DNA populations free and bound to the nanoparticle (see Scheme 1 and Equations (1) and (2)):

(1) $$[DNA]=\frac{1}{1+K^{Au@16-Ph-16\;/DNA-Dauno}[AuNPs]}\times C_{DNA}$$

(2) $$[DNA/AuNPs]=\frac{K^{Au@16-Ph-16\;/DNA-Dauno}[AuNPs]}{1+K^{Au@16-Ph-16\;/DNA-Dauno}[AuNPs]}\times C_{DNA}$$

Accordingly, the observed fluorescence at a fixed wavelength, I_λ_, would be given by Equation (3):

(3) $$I_{\lambda }=\;\frac{I_{F}+I_{B}K^{Au@16-Ph-16\;/DNA-Dauno}\left[\mathrm{AuNPs}\right]}{1+K^{Au@16-Ph-16\;/DNA-Dauno}\left[\mathrm{AuNPs}\right]}$$

The results of fitting the fluorescence experimental data to Equation (3) are given in Table 4, a mean value of K^Au@16-Ph-16/DNA–Dauno^ = 5.1 × 10^7^ (M^−1^) was obtained in water solution, which corresponds to a free energy of binding of −44.0 kJ/mol.

The obtained value of the equilibrium binding constant is presumably consistent with a groove-type biopolymer–nanoparticle binding mode, which generally provides higher interaction constant values between 10^7^ and 10^8^ M^−1^ [54]. However, more direct structural evidence will be needed to confirm or refute this hypothesis. Similar values were obtained for the interaction of Au@thiopronine nanoparticles with the same type of long-chain DNA used in this study, where the binding was promoted by the formation of hydrogen bonds between the hydrophilic groups of thiopronine and the interior of the DNA bases [55]. In this case, the 16-Ph-16 surfactant that stabilizes the gold nanoparticle could interact both electrostatically with the phosphate groups of the DNA and through hydrophobic interactions with the bases, stabilizing the binding. A high free energy value of the nanocomplex formation guarantees its stability in solution and its use as an efficient nanocarrier for the drug [56], maintaining its structure and drug payload during transport and release.

### 3.3. Circular Dichroism (CD) Spectroscopy: DNA/Dauno and Au@16-pH-16/DNA–Dauno Study of Conformational Changes

**Figure 2:**
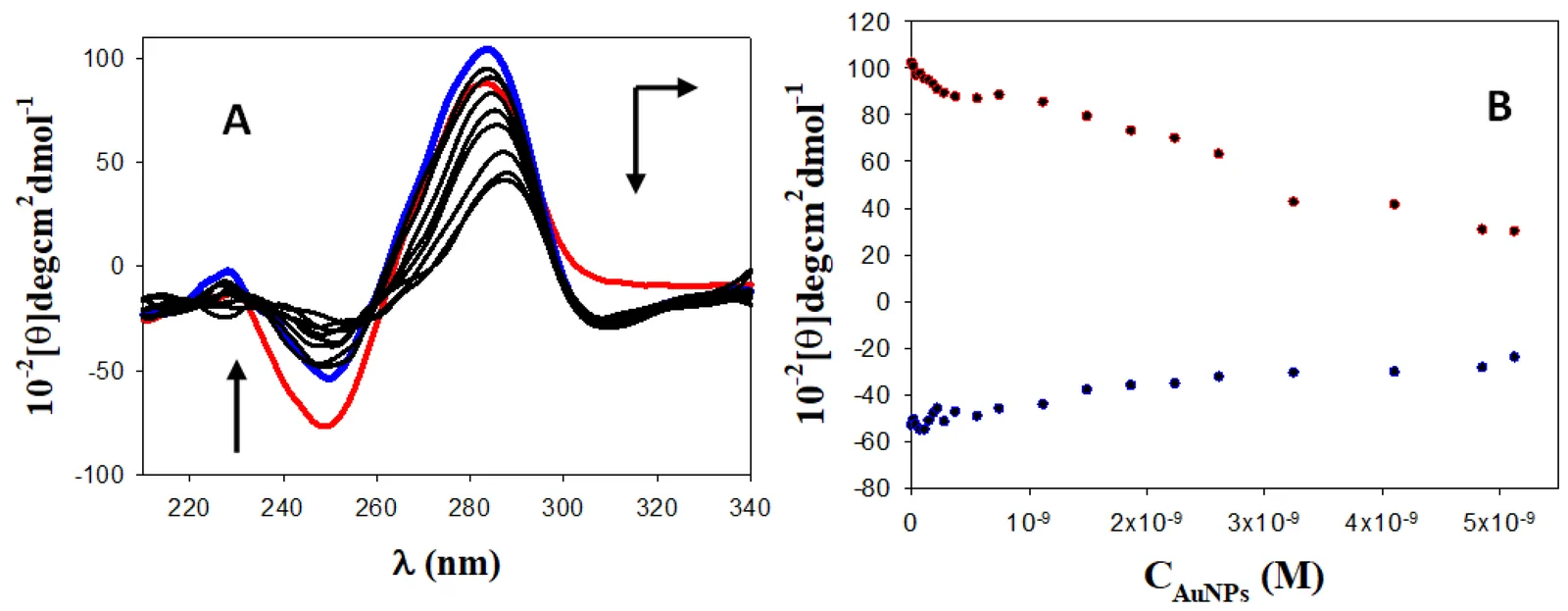
CD titration of Au@16-Ph-16/DNA–Dauno system measured in water solution at T = 25.0 °C; C_DNA_ = 100 μM and C_Dauno_ = 10 μM. (**A**) Curves correspond to C_AuNPs_: (●) 0 M in the absence of Dauno drug; (●) 0 M in the presence of Dauno drug; (●) arrows indicate change at 0.186, 0.747, 1.49, 2.24, 2.61, 4.11, 4.85, and 5.18 nM. (**B**) Figure corresponds to the plot of maximum (●) and minimum (●) CD molar ellipticity of the Au@16-Ph-16/DNA–Dauno system at different C_AuNPs_ concentrations.

Based on the DLS and fluorescence results, it can be assumed that the strong interaction of the nanoparticle with the DNA-D complex could induce the compaction of the biomolecule, generating small Au@16-Ph-16/DNA–Dauno nanocomplexes. To confirm or refute this hypothesis, complementary spectroscopic and structural techniques were employed. Therefore, CD spectra were performed in the absence and presence of gold nanoparticles to explore the nature of the conformational changes that occur upon Au@16-Ph-16 binding (Figure 2).

Figure 2A shows a CD spectrum (red color) that corresponds to DNA in the right-handed B form, where the positive and negative peaks at 280 nm and 249 nm are, due to the stacking interactions between DNA bases and the asymmetric superhelix structure of the biopolymer [57]. The interaction of the drug daunomycin produces an increase in the intensity of the positive CD spectrum band, indicating a perturbation of the bases’ stacking interactions, along with a decrease in the negative band (blue color), indicating that the Dauno–drug modifies DNA structure by helix intercalation [58]. Moreover, analyzing the CD spectra in the ICD region through Dauno interaction, the appearance of a negative peak around 300 nm can be observed which is consistent with intercalation mode binding [59]. Upon Au@16-Ph-16 addition to the DNA-D complex (black color spectra, arrow’s sense) a weaker change in the intensities of the positive and negative CD peaks of DNA is followed by a stronger decrease in the intensity of both CD bands which is accompanied by a right shift in the positive band (see Figure 2A). The initial behavior observed nanoparticle concentrations of around 0.747 nM, consistent with the possibility of nanoparticle binding to the DNA groove, as previously suggested in Section 2, since it is known that CD changes induced by groove binders are less intense than those produced by intercalators [60]. The second behavior is indicative of DNA denaturalization, double helix unwinding and DNA compaction induced by gold nanoparticles [61]. However, Figure 2A,B shows how the decrease in the positive band and the shift in the positive peak to a higher wavelength is more noticeable when the nanoparticle concentration exceeds 2.2 nM, which could indicate the existence of aggregation processes in previously compacted nanosystems [62].

On the other hand, the relative contribution of the DNA’s 275 band associated with the A and G purine base content compared to the 245 nm band, which is associated with the C and T pyrimidine bases of DNA[63], is depicted in Figure S9. In this figure, a ratio between the changes in molar ellipticity at 280 and 249 nm was quantified and represented versus the Au@16-Ph-16 concentrations. As this ratio decreases sharply with nanoparticle concentration, this behavior is indicative of a high contribution from the positive band, consistent with the biomolecule compaction process and the transition to a C-DNA-type structure.

Thus, based on CD spectra changes, the optimal relative Au@16-Ph-16/DNA concentration for preparation of Au@16-Ph-16/DNA–Dauno nanocomplexes in Table 1 was R = C_Au@16-Ph-16_/C_DNA_ = 2.2 × 10^−5^. That is, by establishing this molar ratio, smaller nanostructures would be obtained, avoiding possible aggregation processes between them. In any case, this postulated tendency toward aggregation must be verified in greater depth using AFM and DLS techniques.

### 3.4. Atomic Force Microscopy (AFM) and DLS Experiments

**Figure 3:**
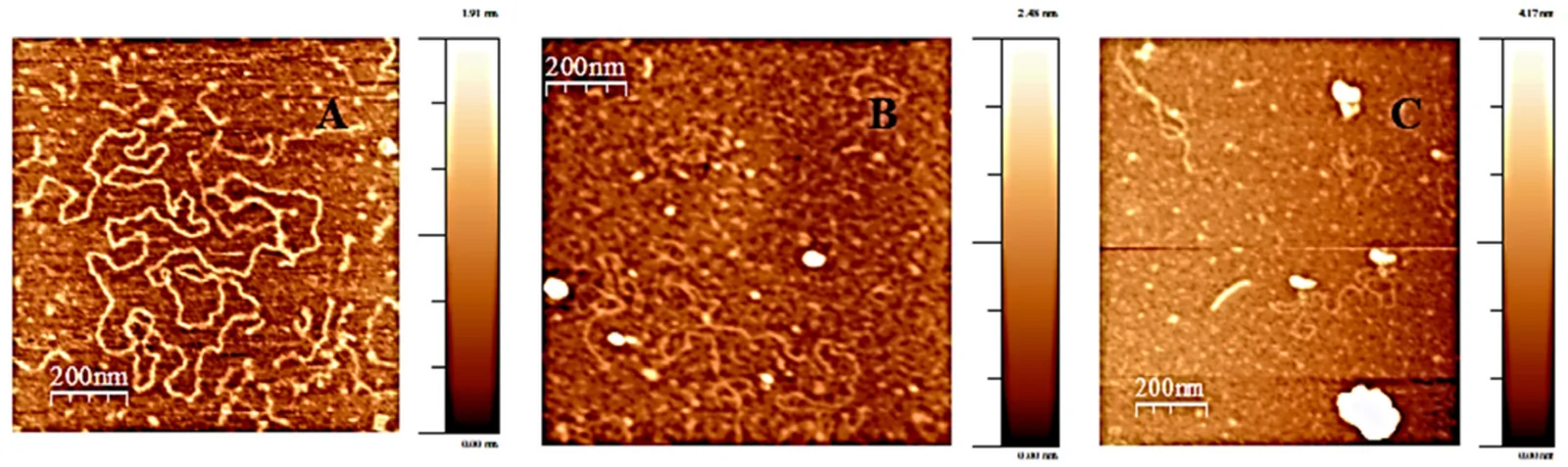
AFM topography images of DNA and DNA/Dauno complex adsorbed on 3-aminopropyltriethoxilane (APTES)-modified mica surface (C_DNA_ = 0.3 μM). (**A**) DNA, C_Dauno_ = 0 µM. (**B**,**C**) DNA/Dauno complexes C_Dauno_ = 0.03 µM.

Atomic force microscopy (AFM) is a powerful imaging technique that allows different biomolecules, including DNA, to be characterized at the structural level with nanometric resolution [64]. Figure 3A shows AFM topographic image of double-stranded calf thymus DNA adsorbed onto an APTES modified mica surface in the absence of Dauno and gold nanoparticles. In this image the biomolecule is in extended coil conformation. However, when drug daunomycin was added to the biomolecule in a molar ratio 1:10, X = C_Dauno_/C_DNA_ = 0.10, morphological changes from the elongated coil state to partially compacted DNA structures can be seen, which indicate the influence that Dauno has on the DNA structures. Figure 3B,C show intermediates of the DNA compaction process where both elongated DNA of a smaller size and in a lower proportion than that observed in the absence of the drug, and compacted DNA structures in globular form, thick fibers, and flower-like structures containing DNA loops on the outside can be observed. However, special attention should be paid to avoiding, as far as possible, different types of artifacts that may arise in AFM, such as drying artifacts [65].

**Figure 4:**
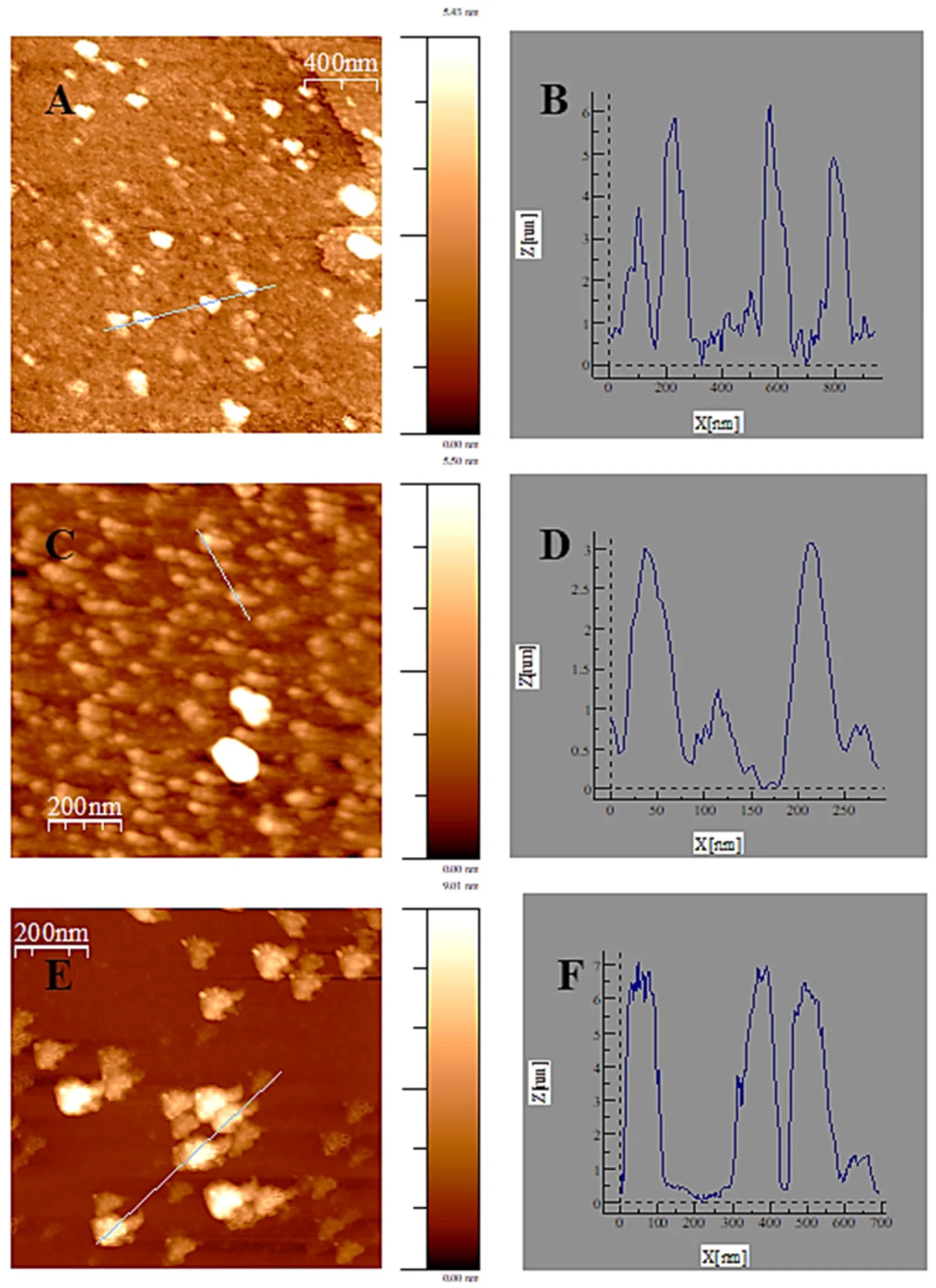
AFM topography images of Au@16-Ph-16/DNA–Dauno nanocomplexes adsorbed on 3-aminopropyltriethoxilane (APTES)-modified mica surface (C_DNA_ = 0.3 μM, C_Dauno_ = 0.03 μM) under different reaction conditions. (**A**) Intermediates of compaction; C_Au@16-Ph-16_ = 0.00066 nM. (**C**) C_I_ nanocomplexes at a compact stage; C_Au@16-Ph-16_ = 0.0066 nM. (**E**) Aggregated form of compacted C_I_ nanocomplexes; C_Au@16-Ph-16_ = 0.066 nM. (**B**,**D**,**F**) Cross section analysis along the selected line in blue colour for (**A**,**C**,**E**), respectively.

In AFM studies of the DNA/Dauno complex carried out in the presence of nanoparticles, it can be easily seen how small concentrations of nanoparticles promote a conformational change towards more compact structures. In this way, in Figure 4A,B, densely packed compact structures were observed, distributed homogeneously along the mica plate. Height/length histograms in z and X-Y direction in Figure S10A,B show particles of (110 ± 16) nm long (X-Y direction) and (3.8 ± 0.5) nm high (z direction). However, this conformational change was not fully accomplished until a nanoparticle concentration that was 10 times higher was reached, which corresponds to a molar ratio of R = C_Au@16-Ph-16_/C_DNA_ = 2.2 × 10^−5^ in agreement with the results from CD studies (see Figure 4C,D).

Note that for this concentration, the average size of the nanocomplexes is significantly smaller, as shown in Figures S10C-D. Thus, particles of (61 ± 5) nm in X-Y direction and (3.0 ± 0.5) nm in height can be visualized, which is indicative of a sgolution state compaction of this sample. Note that the visualization of only a few isolated nanocomplexes larger than 100 nm was insignificant considering the different areas explored on the surface of the mica. Finally, when we compared the structures presented in Figure 4C,D (at C_Au@16-Ph-16_ = 0.0066 nM) with those visualized in Figure 4E,F (at C_Au@16-Ph-16_ = 0.0660 nM), the AuNPs-induced nanocomplexes aggregation was evident. That is, the increase in nanoparticle concentration causes the formation of aggregate structures. It generates the formation of aggregate structures of (255 ± 26) nm in X-Y direction, which can be visualized together with nanosystems smaller than this, measuring (100 ± 4) nm (see Figure S10E,F). Note that the height is also greater, (6.5 ± 1.2) nm, evidencing the aggregation phenomena in z direction. Hence, the origin of the formation of Au@16-Ph-16/DNA–Dauno aggregates is presumably due to the direct interaction between Au@16-Ph-16/DNA–Dauno and free Au@16-Ph-16. Similar behavior was observed in the AuNPs/ss-DNA and AuNPs/ds-DNA systems, where both NaCl and the addition of Au@thiopronine at higher concentrations promoted interconnections between the nanoparticles and, consequently, aggregation of the nanosystem [66].

To verify that these phenomena of compaction and aggregation observed on mica were also present in solution, complementary DLS experiments were carried out under the same experimental conditions. Thus, the results in Figure S8 showed similar behavior for the DNA–Dauno system when increasing amounts of Au@16-Ph-16 nanoparticles were added. That is, at C_Au@16-Ph-16_ = 0.00066 nM concentrations, the average hydrodynamic diameter of the nanocomplexes was (124 ± 14) nm. Subsequently, the addition of a nanoparticle concentration of 0.0066 nM produced small nanocomplexes (59 ± 11) nm, evidencing the full compaction of the system in accordance with AFM and CD results. Finally, adding a 10-fold higher concentration of nanoparticles resulted in nanocomplexes with a bimodal size distribution, evidencing the aggregation process. Accordingly, 80.5% of the nanocomplexes were slightly larger than the previous ones (97 ± 11) nm, while the remaining 19.5% corresponded to nanocomplexes of a considerably larger size (397 ± 25) nm, which correspond to the aggregation of different nanosystems with each other. It should be noted that this result is consistent with the AFM study, in which aggregated nanostructures formed by at least 3–4 nanocomplexes joined together can be visualized from Figure 4E,F. Therefore, the discussed AFM and DLS data strongly support the compaction/aggregation initial formulated hypothesis in Section 3.3.

Therefore, considering the results of this structural, spectroscopic, and hydrodynamic study of the interaction between the DNA–Dauno complex and Au@16-Ph-16 nanoparticles, it can be established that the optimal compaction conditions are those that verify a molar ratio, R = 2.2 × 10^−5^. Thus, the C_1_, C_2_ and C_3_ nanosystems shown in Table 1 (see Section 2.1.3) correspond to these previously established conditions. These formulations will be used in subsequent cell viability studies to evaluate their effectiveness as a potential treatment for childhood leukemia. Thus, first, the compacted C_1_, C_2_ and C_3_ nanosystems will be administered at the cellular level to evaluate their effectiveness. On the other hand, this study will be completed with the addition of Au@16-Ph-16 nanoparticles at the concentration necessary to induce aggregation between the different nanosystems. In this way, the effect of subsequent aggregation on the treatment will also be evaluated. In this regard, previous studies have revealed how the aggregation of nanoparticles within cells improved both the retention time and cellular uptake of nanoparticles in tumors, thereby increasing their efficacy as a drug in cancer NIR photothermal therapy [67]. In another work, the strategy of activating NK cell-based immunochemotherapy was proven via a pH-responsive self-aggregated nanoparticle for the codelivery of chemotherapeutic doxorubicin (DOX) and the inhibitor SIS3, resulting in an inhibition of melanoma tumor growth [68]. It is important to note that this work introduces a significant methodological innovation, namely, the aggregation of the compacted nanosystem is induced by simply adding small nanoparticles to the system, which can be easily internalized at the cellular level due to their small size and high charge. All of this is achieved without the need for photothermal methods or the use of codelivery inhibitors, expanding its potential for application in different cancer therapies.

### 3.5. In Vitro Biocompatibility of AuNPs and Nanosystems Viability Assay

**Figure 5:**
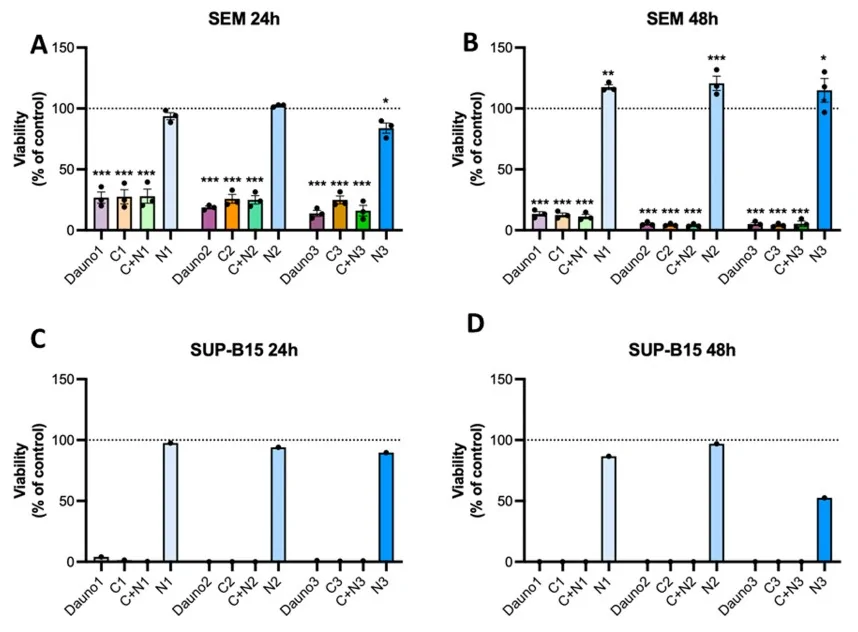
Graphical representation of cell viability of SEM (**A**,**B**; *n* = 3) and SUP-B15 (**C**,**D**; *n* = 1) cells after 24 and 48 h of incubation with Dauno free or bound to gold nanosystems. Values represent percentage of proliferation of cells treated with the different experimental conditions with respect to control cells. Asterisks indicate statistically significant differences with respect to control cells (* *p* < 0.05, ** *p* < 0.01, *** *p* < 0.001).

As mentioned above, this study will explore the effect of nanocomplex aggregation induced by the aggregation of well-dispersed nanoparticles (N_I_) to a previously internalized compacted nanosystem (C_I_) in cancer therapy. This possible synergistic effect derived from the use of combined nanosystems (C_I_ + N_I_) will be compared with that exerted by the compacted nanosystem itself (C_I_). Accordingly, Figure 5 shows how C_I_, N_I_ + C_I_, and Dauno formulations significantly reduced cell viability of both SEM and SUP-B15 cell lines at 24 and 48 h independently of the daunomycin concentration. Remarkably, we observed an even more potent effect on SUP-B15 cells, suggesting that these cells may be presumably more sensitive to Daunomycin treatment, showing almost no fluorescence value in response to daunomycin, which may indicate a potent cytotoxic effect.

**Figure 6:**
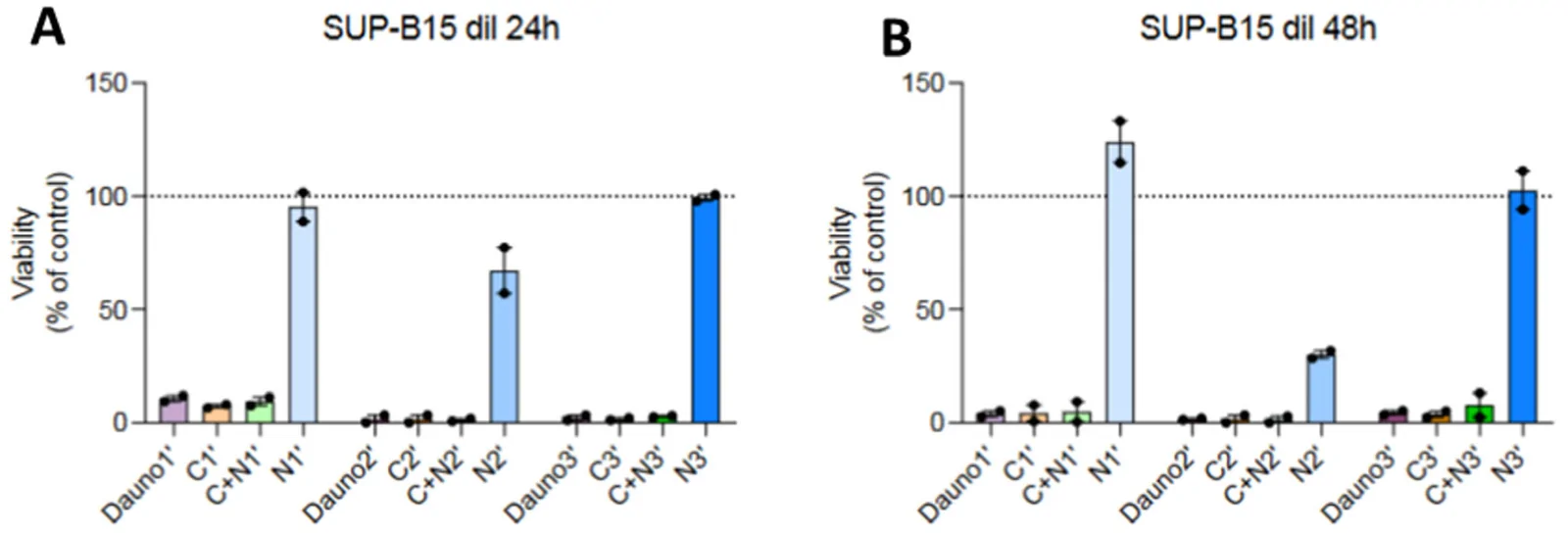
Graphical representation of cell viability SUP-B15 cells (*n* = 3), for concentration diluted, after 24 h (**A**) and 48 h (**B**) of incubation with daunomycin free or bound to gold nanosystems. Values represent percentage of proliferation of cells treated with the different experimental conditions with respect to control cells.

Consequently, the nanosystems were modified to reduce the concentration of Dauno. The result was that the diluted compounds significantly reduced the viability of SUP-B15 at 24 and 48 h of incubation. (Figure 6 and Table S1), with a high significance of *p* < 0.001 in the statistical analysis carried out, the most effective being those corresponding to concentration 2 and 3 in the tests of both the treatments with Au@16-Ph-16/DNA–Dauno as well as Au@16-Ph-16/DNA–Dauno + Au@16-Ph-16/DNA for 24 h and especially the concentration. Interestingly, both C_I_′ and N_I_′ + C_I_′ formulations provoked a similar effect as Daunomycin as a single agent, indicating that nanoparticle aggregation does not potentially alter its effectiveness, while no additive effect was found in N_I_′ + C_I_′ compared to C_I_′. Remarkably, gold nanoparticles by themselves (N_I_′) did not markedly affect cell viability of SEM and SUP-B15 cells apart from the case of N_2_′ treatment in SUP-B15 cells, where the viability at 24 and 48 h was significantly reduced, reaching its maximum at 48 h. These solutions are used to conduct internalization studies due to the cell destruction caused by C_3_ and C_3_ + N_3_ concentrations. Therefore, it is confirmed that these are indeed the ones that are effective for proper treatment.

Since AuNPs, do not produce damage in the cells, it demonstrates the vehiculizing and non-toxic role of the AuNPs used, due to their adequate size and their properties to exert this effect [20,21,69,70]. The enhancing effect of the addition of AuNPs to the nanosystems can be observed at 24 h, for the C_3_ concentration, being similar for the rest of the concentrations and times.

In previous studies, it has been observed that certain nanosystems target tumor tissue more efficiently by taking advantage of the specific characteristics of cancer cells [21]. This phenomenon is repeated in our study, where similar behavior is observed. In particular, the results of treatments with C_3_ + N_3_. This highlights the high effectiveness of C_3_ + N_3_ nanosystems as anticancer agents, outperforming standard treatment in terms of precision and efficiency in eliminating tumor cells.

Dauno administered in its free form can exhibit low selectivity towards the tumor and cause significant side effects, such as acute cardiotoxicity and bone marrow depression. The development of drug transport systems has corrected the limitations associated with drug delivery in its free form [71,72].

Thus, these results suggest that Au@16-Ph-16/DNA–Dauno nanocomplexes, both combined with N_I_ (C_I_ + N_I_ treatment) and free in solution (C_I_ treatment) constitute potential biocompatible therapies for ALL of B cells patients, unlike further analyses using non-malignant control cell models.

### 3.6. Internalization of Au@16-Ph-16 and Au@16-Ph-16/DNA–Dauno Nanosystems

**Figure 7:**
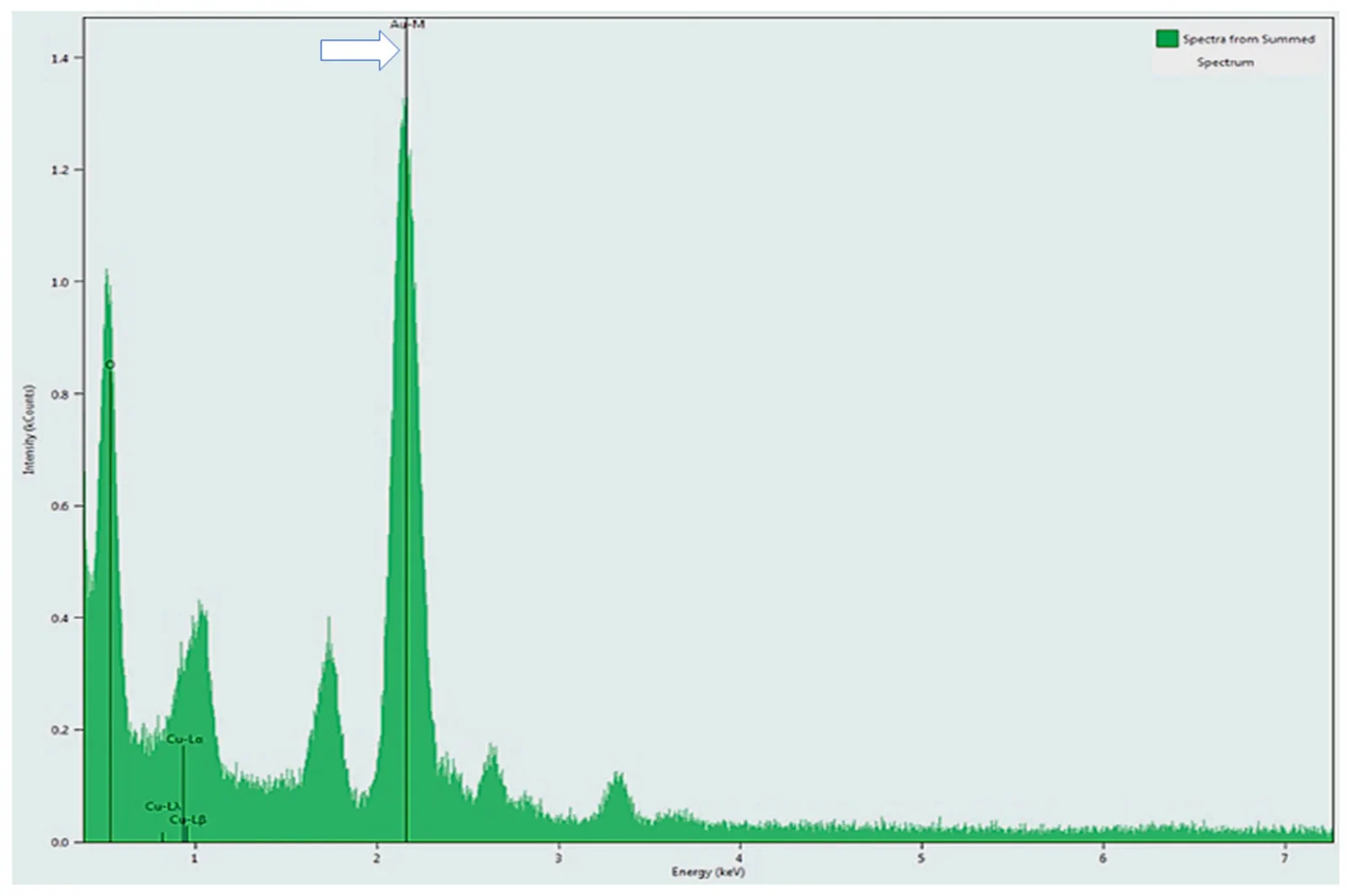
EDS spectrum of Au@16-Ph-16 nanoparticles. Elemental mapping results indicate the distribution of gold element on the particles. Bear in mind that the presence of copper ion is due to the use of copper grid coated with a carbon film needed for TEM measurement. The arrow indicates the golden peak.

The EDS described in the corresponding section is performed, showing the presence of gold in the analyses performed (Figure 7). The figure shows the microanalysis performed in TEM TALOS. Note that other elements besides gold are also detected. That is, copper which is part of the components of the grid, part of the products used to fix the cells, and some elements such as carbon, which are part of the organic material.

**Figure 8:**
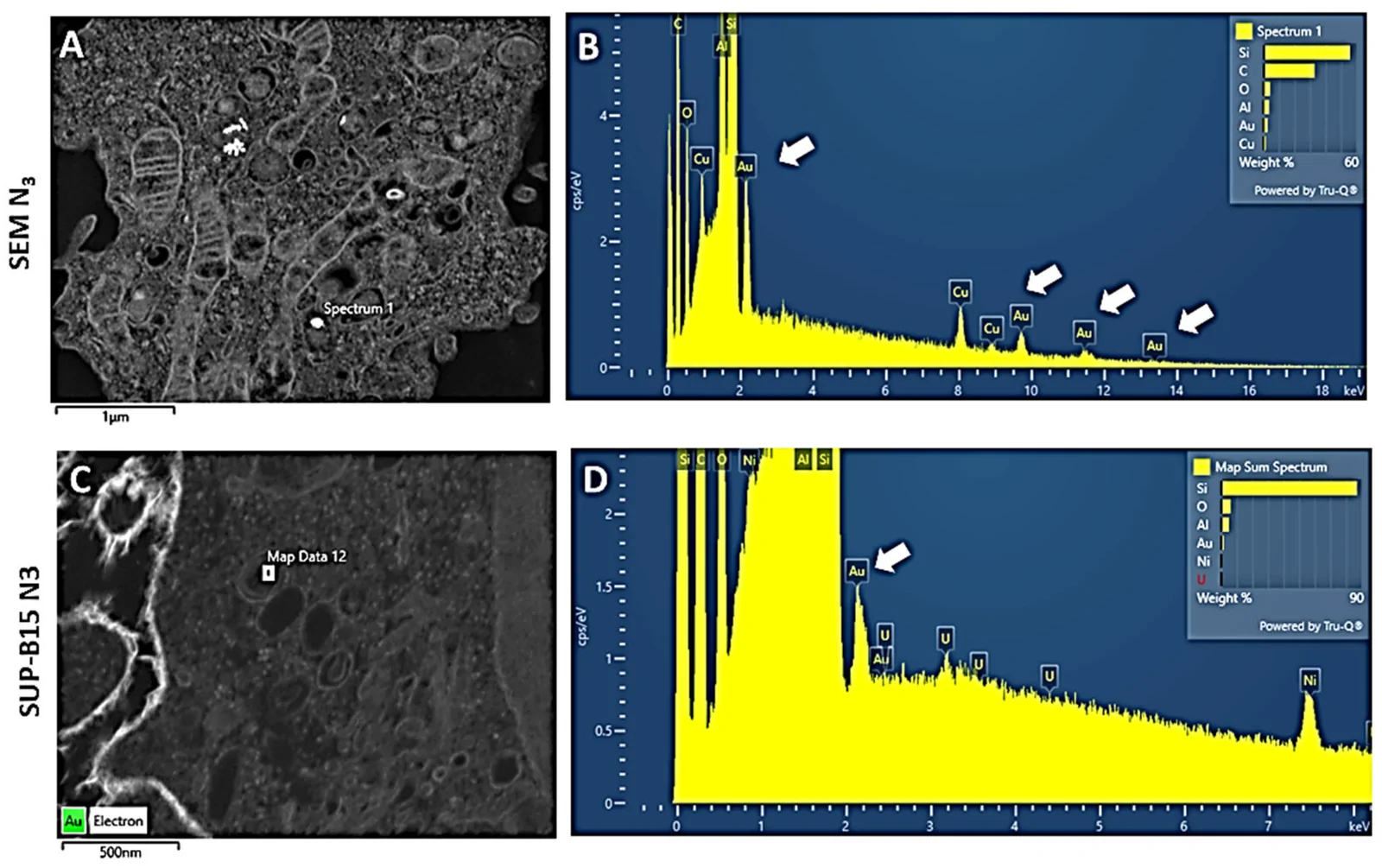
Analysis EDS with a laser scanning confocal microscope on an ultrathin section of fixed cells, showing the presence of gold from nanosystems inside the cells. (**A**) shows the scanning electron microscope image of cells where the spectrum is performed and the corresponding spectrum (**B**). (**C**) shows the scanning electron microscope image of SEM cells where the spectrum is performed and the corresponding spectrum (**D**). In both EDS, the presence of Au and other elements resulting from the preparation is demonstrated. The arrow indicates the golden peak.

On the other hand, the results performed with a laser scanning confocal microscope on cell preparations show the presence of gold inside the cells (Figure 8), both in SEM cells (Figure 8A,B) and in SUP-B15 cells (Figure 8C,D). Based on the results obtained, it can be verified that different elements result from the fixation and contrast treatment, with the presence of gold belonging to the nanosystem.

**Figure 9:**
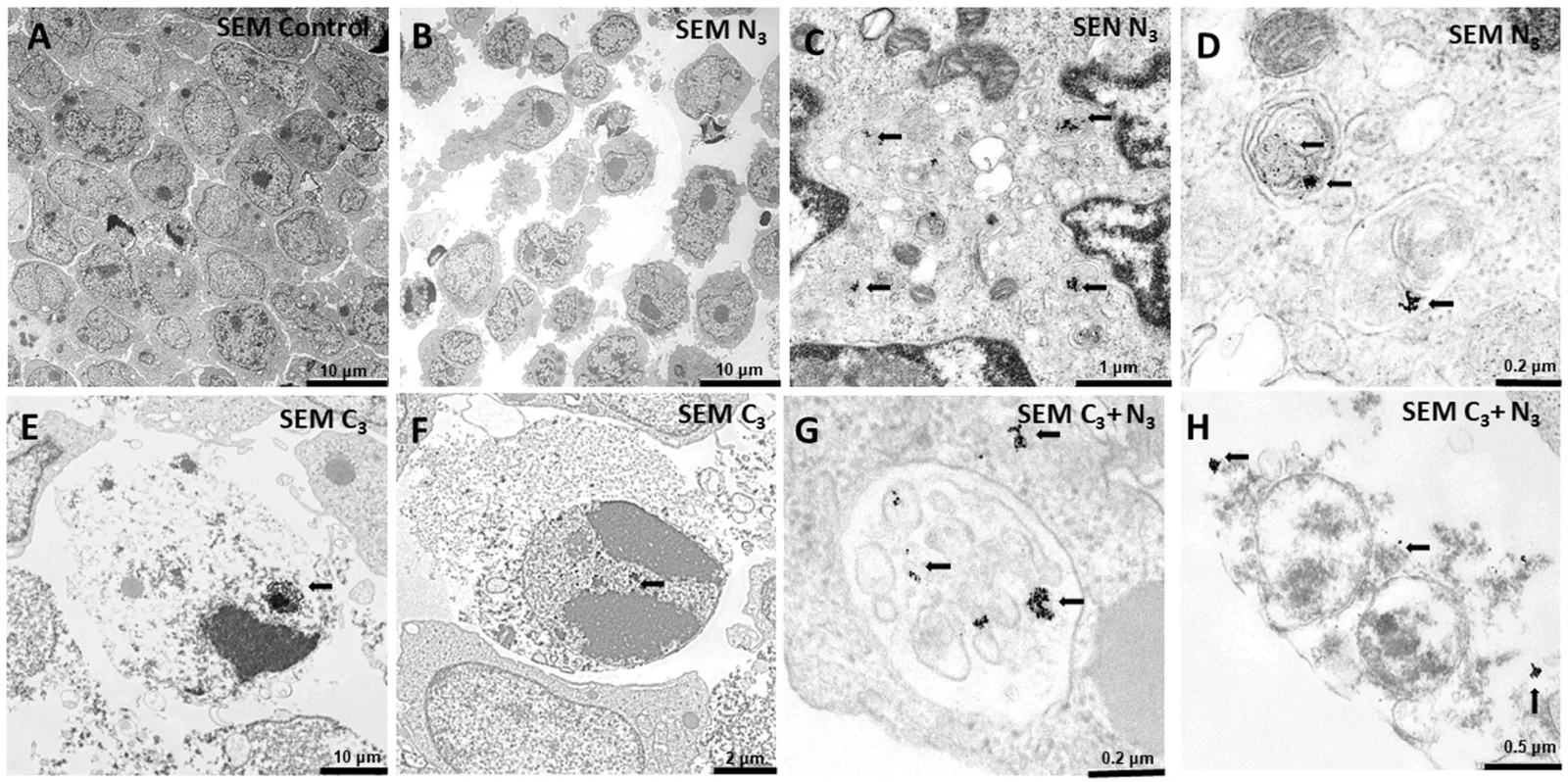
Transmission electron micrograph (TEM) in SEM cells, of control cells without treatment with Au@16-Ph-16/DNA–Dauno (**A**), compared to Au@16-Ph-16 (N_3_) (**B**–**D**), Au@16-Ph-16/DNA–Dauno (C_3_) (**E**–**F**), and Au@16-Ph-16/DNA–Dauno + Au@16-Ph-16 (C_3_ + N_3_) (**G**–**H**) at 24 h. Arrows indicate the presence of nanoparticles (**A**–**H**).

**Figure 10:**
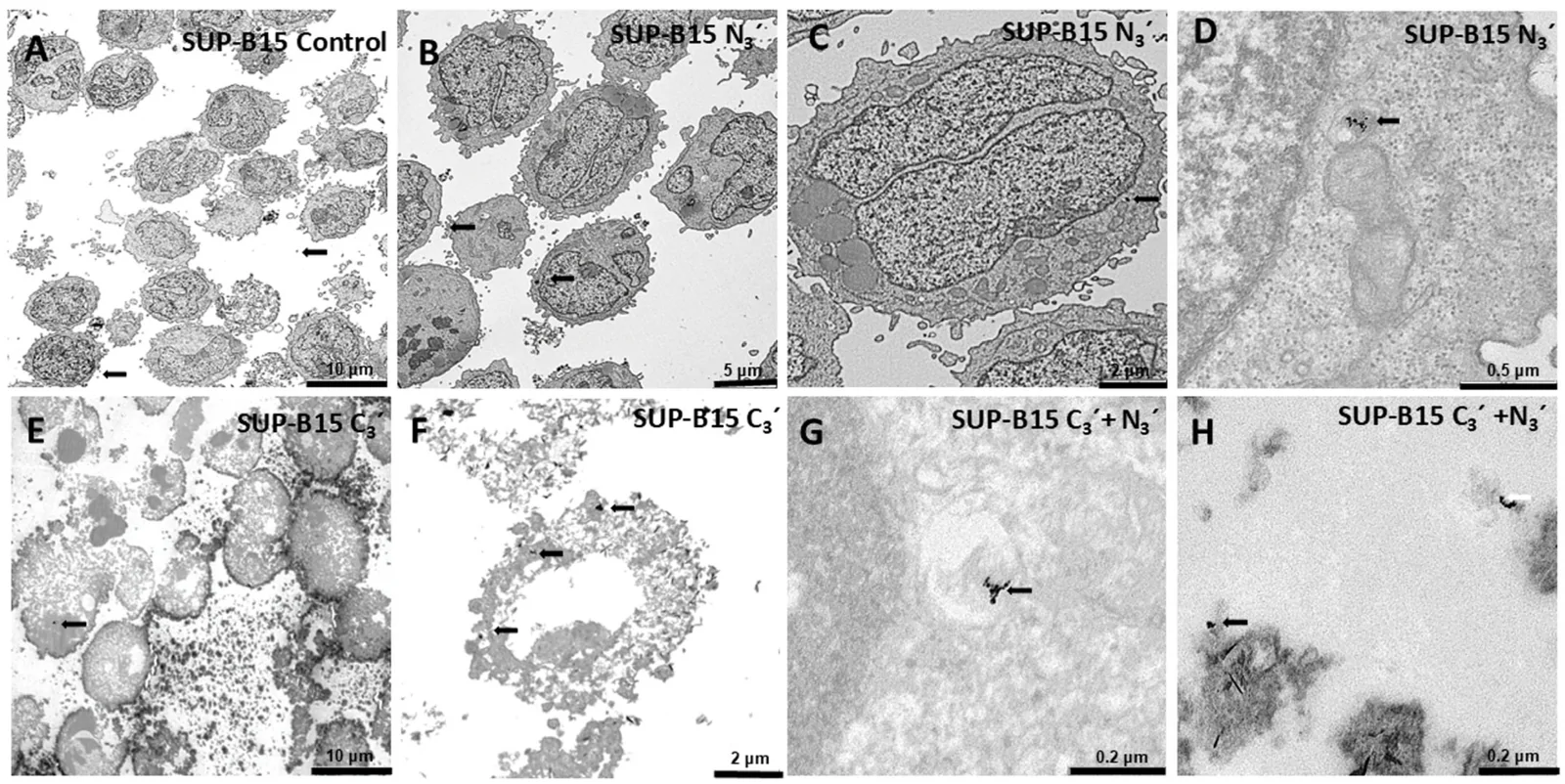
Transmission electron micrograph (TEM) in SUP-B15 cells, of control cells without treatment with Au@16-Ph-16/DNA–Dauno (**A**), compared to Au@16-Ph-16 (N_3_′) (**B**–**D**), Au@16-Ph-16/DNA–Dauno (C_3_′) (**E**,**F**) and Au@16-Ph-16/DNA–Dauno + Au@16-Ph-16 (C_3_′ + N_3_′) (**G**,**H**) at 24 h. Arrows indicate the presence of nanoparticles (**A**,**H**).

**Figure 11:**
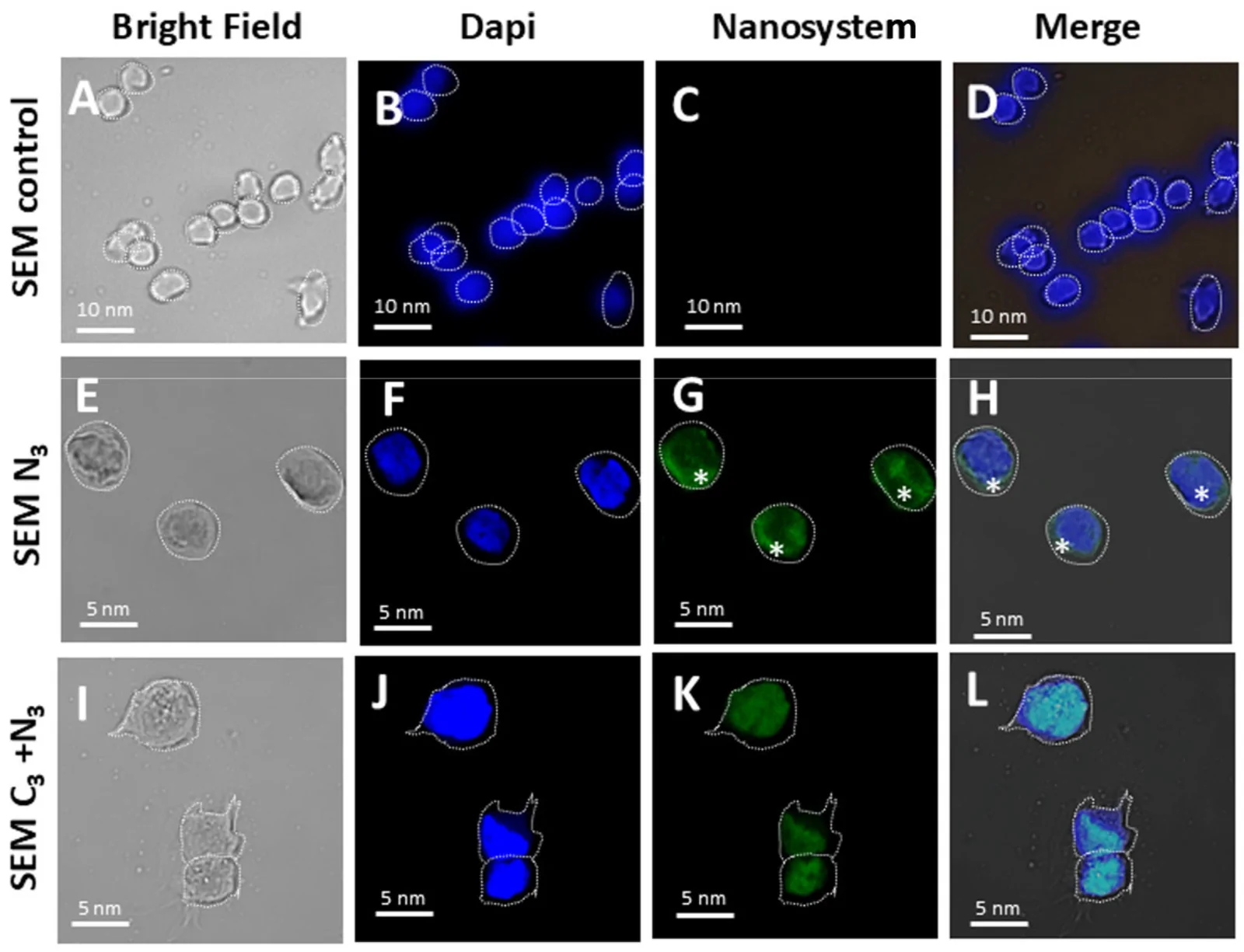
Confocal microphotographs showing cells SEM treated for 24 h. First column shows transmitted light microphotography; second column, DAPI-labeled nuclei; third column, green fluorescently labeled with nanoparticles or/and nanosystems; and fourth column, merge. Control cells without treatment (**A**–**D**). Cells treated with Au@16-Ph-16 (N_3_′) (**E**–**H**), and cells treated with Au@16-Ph-16/DNA–Dauno C_3_ and Au@16-Ph-16 (N_3_), (C_3_ + N_3_) (**I**–**L**). Asterisks indicate nanoparticles outside the cell.

**Figure 12:**
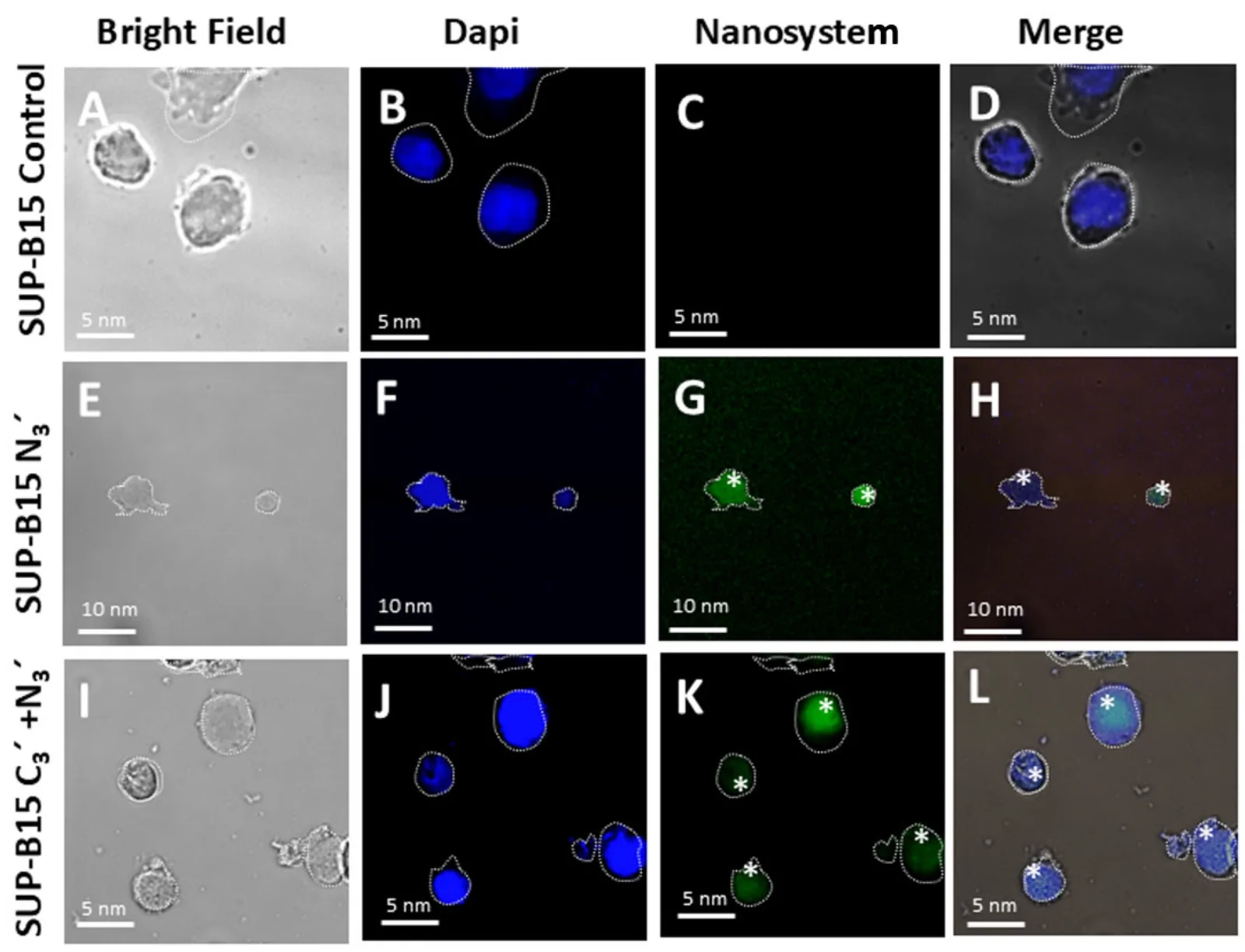
Confocal microphotographs showing cells SUP-B15 treated for 24 h, in their diluted concentration. First column shows transmitted light microphotography; second column, DAPI-labeled nuclei; third column, green fluorescently labeled with nanoparticles or/and nanosystems; and fourth column, merge. Control cells without treatment (**A**–**D**). Cells treated with Au@16-Ph-16 (N_3_′) (**E**–**H**), and cells treated with Au@16-Ph-16/DNA–Dauno C_3_′ and Au@16-Ph-16 (N_3_′), (C_3_′ + N_3_′) (**I**–**L**). Asterisks indicate nanoparticles outside the cell.

In addition to EDS analysis, TEM (Figure 9 and Figure 10) and laser scanning confocal microscope studies (Figure 11 and Figure 12) were performed to demonstrate the entry of both Au@16-Ph-16 nanoparticles and Au@16-Ph-16/DNA–Dauno nanosystems into the cells. These microscopy techniques allow the visualization of the organelles and can be used to verify the presence of AuNPs inside the cells. In this case we show the results of both SEM cells (Figure 9) and SUP-B15 cells, in their diluted concentration (Figure 10), for their effectiveness and at 24 h to ensure their visualization, since at 48 h there is a lot of cell destruction. In the control study performed on SEM cells (Figure 9) without any treatment, we observed that the cells present typical organelles of the type to which they belong (Figure 9A). In cells treated with N_3_ (Figure 9B,D), dense rounded structures are observed, marked with arrows, which are compatible with the AuNPs nucleus. The presence of N_3_ can be clearly observed in vesicles that are part of the usual membrane traffic in cell metabolism, which is very high in these cells, and hence we can observe numerous mitochondria that provide the energy necessary for their metabolism. Gold nanoparticles are suitable for conjugation with a variety of biologically active groups, being good elements for their application in biomedicine ranging from diagnostics, targeted drug delivery, among others, including electron microscopy marker detection [20,21,69,70]. In this case, and as has also been demonstrated in toxicity studies, N_3_ nanoparticles do not appear to cause damage to cells; they simply have an easy entry into the cells, given their adequate size.

However, in cells treated with C_3_ (Figure 9E,F), these dense bodies compatible with the gold nuclei can be observed, as well as how the process of cell degradation is evident with membrane degradation and loss of cellular material. This effect takes place after the release of the different compounds of the nanosystems (DNA and Dauno) inside the cell, with the drug acting directly on the biosynthetic machinery of the cell and not allowing the defensive response. It is well known that AuNPs are vehicles for different substances inside microorganisms and cells. The process is repeated in cells treated with the combined treatment C_3_ + N_3_, which is carried out by subsequently adding the N_3_ nanoparticle to the previously internalized C_3_ nanocomplex. Consequently, Figure 10G shows that the internalization process is clearly observed both at the vesicular level and in free form inside the cell. It should also be noted that nanoparticles in this type of treatment (C_3_ + N_3_) are mainly found in the form of aggregates inside cells, which is consistent with what has been previously observed in solutions based on AFM, DLS, and CD studies. Moreover, Figure 10H shows the uptake of these elements through the membrane and the cellular debris resulting from cellular disintegration, which demonstrates the high effectiveness of the combined treatment in just 24 h of action.

On the other hand, Figure 10 shows the results obtained to demonstrate the internalization of N_3_′ nanoparticles and C_3_′ nanosystem in the cellular interior of SUP-B15 cells after 24 h of treatment. Figure 10A shows cells without any treatment where the morphology of these cells is easily observed. From Figure 10B–D, the internalization of N_3_′ nanoparticles into vesicles was clearly observed with dense bodies compatible with the gold nuclei. The highly destructive effect of both C_3_′ and the combined C_3_′ + N_3_′ nanosystems is evident from Figure 10E–H. In particular, the effect of the C_3_′ nanosystems (Figure 10E,F), produces the degradation of the biosynthetic machinery of the cells, affecting the destruction of their organelles and cellular emptying. Likewise, the combined C_3_′ + N_3_′ treatment produces separation of the components and direct action of the drug inside the cells, causing irreparable damage and therefore cell death (Figure 10G,H). Once again, these figures show the nanoparticles in aggregate form, demonstrating how the aggregation process inside the affected cells enhances the effectiveness of the treatment.

Additionally, to complete the internalization studies, several confocal microscopy images were collected, showing the presence of nanoparticles inside the cells, confirming the TEM results. The results are shown for both SEM cells (Figure 11) and SUP-B15 cells at their diluted concentration (Figure 12).

In the first column of Figure 11, you can see the silhouettes of the SEM cells in bright field (Figure 11A,E,I). The second column shows the cell nuclei stained with DAPI (Figure 11B,F,J), and in the last column you can see the merge of the previous ones. The first row (Figure 11A–D) shows control images for SEM cells, where only the nucleus shown in blue is positive (Figure 11B,D). The second row shows SEM cells treated with N_3_ (Figure 11E–H), where we can see the entry of nanoparticles into the cell interior in green (Figure 11G,H). The third row (Figure 11I–L) shows SEM cells treated with C_3_ + N_3_, demonstrating that internalization occurs (Figure 11K,L).

In Figure 12, we can see silhouettes of SUP-B15 cells in bright field (Figure 12A,E,I). The second column shows cell nuclei stained with DAPI (Figure 12B,F,J), and the last column shows a merge of the previous images. The first row (Figure 12A–D) shows control images for SUP-B15 cells, where only the nuclei shown in blue is positive (Figure 12B,D). The second row shows SUP-B15 cells treated with N_3_′ (Figure 12E–H), where we can see the entry of nanoparticles into the cell interior in green (Figure 12G,H). The third row (Figure 12I–L) shows SEM cells treated with C_3_′ + N_3_′, demonstrating that internalization occurs (Figure 12K,L).

These results demonstrate the internalization of the nanoparticles and nanosystems tested. On the other hand, the particles that internalize in the cells are counted using confocal microscope images. Of the nearly 17,000 particles counted, SEM cells resulted (M ± SD) in a total of 165.1 ± 68.3 particles for N_3_′, 118.2 ± 66.2 for C_3_′ + N_3_′, while for SUP-B15 cells, a total of 62.4 ± 32.6 particles were obtained for N_3_′ and 39.3 ± 34.5 for C_3_′ + N_3_′. These results are coherent considering that the main outcome of this work is the fact that the bare nanoparticle itself (Au@16-Ph-16) at the appropriate concentration can induce the aggregation of the ternary nanosystems once it has entered the cell, further enhancing their anticancer effect. Furthermore, lower amounts are observed in SUP-B15 cells compared to SEM cells. It should be noted that due to the high destruction of SUP-B15 cells at concentration 3, we had to use diluted concentration, as detailed above, to see internalization in this cell type. Possibly, the high destruction and the lack of effect of Au@16-Ph-16 caused the observed difference and the lower number of particles for C_3_′ + N_3_′ indicates cell destruction after treatment.

## 4. Conclusions

The designs of daunomycin nanocarriers with Au@16-pH-16/DNA–Dauno contribute significantly to the efficacy and treatment of childhood leukemia. The C_I_, N_I_ + C_I_, and Dauno formulations significantly reduced the cell viability of the SEM and SUP-B15 cell lines at 24 and 48 h, regardless of the daunomycin concentration. Concentrations 2 and 3 were the most effective at 48 h for SEM cells, and concentrations 2 and 3 were the most effective at 24 h for SUP-B15 cells, with total elimination of SUP-B15 cells at 48 h at all concentrations.

One of the anticancer drugs used for chemotherapy of leukemias is Dauno alone or supplemented with other elements. However, the most serious side effects associated with its use are cardiotoxicity, which may include ventricular fibrillation, myocarditis, heart failure, ventricular tachycardia, left ventricular dysfunction, pericarditis, atrial fibrillation, and other related complications. Using the presented strategy, Dauno transporters have been designed to act directly on cancer cells using two alternatives: (i) the direct use of compacted gold nanosystems containing DNA–Dauno complexes as a vehicle, C_I_ nanocomplexes; and (ii) the combined use of C_I_ nanocomplexes and N_I_ nanoparticles that induces C_I_ aggregation inside the cell, optimizing the effectivity of the treatment. In this way, we achieve the release of the compounds once they have been internalized, thus minimizing adverse effects constituting promising projections in healthcare. In this study we have carefully designed a pattern for the synthesis of nanosystems based on the previous optimization of the optimal condition for DNA–Dauno complexation with Au@16-Ph-16 nanoparticles, condensation, and aggregation for the transport of daunomycin. Due to their high affinity of Au@16-Ph-16 and DNA–Dauno complexes, stable Au@16-Ph-16/DNA–Dauno were obtained with low toxicity and good biocompatibility as demonstrated by different techniques. Vehicles designed to transport Dauno and other similar structural drugs stand out for their potential use as drug carriers in the treatment of childhood leukemias, since (i) they guarantee controlled drug release through the aggregation of the compacted nanosystem via subsequent addition of the nanoparticle (C_I_ + N_I_ treatment); (ii) they also allow for precise dose adjustment (maintaining the optimal ratio of DNA–Dauno/nanoparticle complex concentrations). Thus, in this work, the effectiveness of different N_I_, C_I_, and N_I_ + C_I_ formulations has been tested in different cell lines, varying the amount of drug transported; and (iii) the surface charge, morphology, and size of N_I_ nanoparticles and C_I_ compact nanosystems are optimal for ensuring effective penetration into target cells. The results obtained in this study demonstrate the high stability of this nanosystem, which also shows potent antitumor activity in pediatric B-ALL cell models. Thus, these results suggest that the use of gold nanoparticles to deliver daunomycin could be a relevant therapeutic tool for B-ALL patients, offering encouraging prospects for more effective and safer treatments for childhood leukemia. This innovative and effective drug-delivery nanocarriers for childhood leukemia treatment was made possible through careful selection of the Au@16-Ph-16 aggregator concentration in each specific Au@16-pH-16/DNA–Dauno nanosystem. To advance this line of research, preclinical trials in murine models are crucial. These studies will allow further evaluation of the efficacy and safety of gold nanocomplexes in a more complex biological context and may lay the groundwork for future clinical applications. Validation in animal models will help confirm the therapeutic potential of this strategy and facilitate its potential transition to more effective clinical treatments for childhood leukemia.

## Figures and Tables

**Table 1:** Distinct N_I_ and C_I_ formulations and the corresponding concentrations of the reactants used for preparing them.

Compound	Au@16-Ph 16 Concentration	Compound	Reactant Concentrations
N_1_	C_Au@16-Ph-16_ = 1.19 nM	C_1_	C_Dauno_ = 0.25 µM C_DNA_ = 2.5 µM C_Au@16-Ph-16_ = 0.055 nM
N_2_	C_Au@16-Ph-16_ = 4.78 nM	C_2_	C_Dauno_ = 1.0 µM C_DNA_ = 10.0 µM C_Au@16-Ph-16_ = 0.220 nM
N_3_	C_Au@16-Ph-16_ = 5.60 nM	C_3_	C_Dauno_ = 1.3 µM C_DNA_ = 13.0 µM C_Au@16-Ph-16_ = 0.286 nM

**Table 2:** Zeta potential, mean size, and PDI values of the precursor Au@16-Ph-16 nanoparticles.

Au@16-Ph-16	Zeta Potential (mV)	Mean Size (nm)	PDI
N_1_	30.4 ± 1.5	24.0 ± 3.0	0.016
N_2_	34.0 ± 3.0	25.3 ± 0.4	0.0003
N_3_	38.8 ± 0.5	21.8 ± 0.8	0.0013

**Table 3:** Zeta potential, mean size, and PDI values of distinct Dauno nanocarriers, C_I_ formulations.

Au@16-Ph-16/DNA–Dauno	Zeta Potential (mV)	Mean Size (nm)	PDI
C_1_	−30 ± 2.0	74 ± 6.0	0.007
C_2_	−31 ± 7.0	77± 3.0	0.0015
C_3_	−33 ± 8.0	82 ± 1.0	0.033

**Table 4:** Binding constants of DNA–Dauno complex to Au@16-Ph-16 nanoparticles and the parameters of the fit of data in Figure 1 to Equation (3).

Intensity,λ	K^Au@16-Ph-16/DNA–Dauno^ (M^−1^)	I_F_	I_B_
I_555 nm_	(5.3 ± 0.5) × 10^7^	205.0 ± 2.0	0.17 ± 0.02
I_592 nm_	(4.9 ± 0.7) × 10^7^	152.3 ± 1.8	0.18 ± 0.08

## Data Availability

The original contributions presented in this study are included in the article/supplementary material. Further inquiries can be directed to the corresponding authors.

## Supplementary Materials

The following supporting information can be downloaded at: https://www.mdpi.com/article/10.3390/pharmaceutics17091236/s1, 1: Result of characterization of 16-Ph-16 cationic gemini surfactant; Figure S1: Combined COSY and TOCSY spectra of compound p-16-Ph-16 together with the respective 1H NMR spectrum on both axes and structure of the compound 16-Ph-16 and combined HSQC and HMBC spectra of compound 16-Ph-16; 2: TEM Protocol; 3: confocal microscope protocol; Figure S2: TEM image of free Au@16-Ph-16 gold nanoparticles and size distribution of Au@16-Ph-16 in water; Figure S3: Stability of Au@16-pH-16 from its synthesis to one month; Figure S4: Zeta potential of Au@16-Ph-16 nanoparticles at different Au@16-Ph-16 concentrations; Figure S5: DLS size distribution by number of Au@16-Ph-16 for different formulations; Figure S6: Zeta potential of Au@16-Ph-16/DNA–Dauno compacted nanocomplexes for different formulations; Figure S7: DLS size distribution by number of Au@16-Ph-16/DNA–Dauno compacted nanocomplexes for different formulations; Figure S8: Au@16-Ph-16 dependence on the hydrodynamic diameter of Au@16-Ph-16/DNA–Dauno nanocomplexes; Table S1: Diluted concentrations used for both viability and internalization studies; Table S2: Zeta potential and PDI values of the precursor Au@16-Ph-16 nanoparticles and distinct Dauno nanocarriers, Ci formulation in serum and PBS buffer; Figure S9. Changes in molar ellipticity ratio between 280 nm and 249 nm of DNA versus the gold nanoparticle concentration; Figure S10. Height/length histograms in z and X-Y direction for AFM topographic samples.
