# Supplementary material for: Daunomycin Nanocarriers with High Therapeutic Payload for the Treatment of Childhood Leukemia
Source: Pharmaceutics. 2025 Sep 22;17(9):1236. doi: 10.3390/pharmaceutics17091236 (PMC12473718; doi:10.3390/pharmaceutics17091236)
Supplement: Supplementary file 1 [file pharmaceutics-17-01236-s001.zip › pharmaceutics-3850851-supplementary.pdf]

Daunomycin nanocarriers with high therapeutic payload for the treatment of childhood leukemia

Rosa M. Giráldez-Pérez [1,*,†], Elia Grueso [2,*,†], Antonio J. Montero-Hidalgo [1,3], Cristina Muriana-Fernández [1], Edyta Kuliszewska [4], Raúl M. Luque [1,3,5] and Rafael Prado-Gotor [2]

[1] Department of Cell Biology, Physiology and Immunology, Faculty of Sciences, University of Cordoba, 14014 Cordoba, Spain
[2] Department of Physical Chemistry, Faculty of Chemistry, University of Seville, 41012 Seville, Spain
[3] Maimonides Biomedical Research Institute of Cordoba (IMIBIC); Reina Sofia University Hospital (HURS); 14004 Cordoba, Spain
[4] Chemtra, 47-300 Krapkowize, Poland; edyta.kuliszewska@interia.pl
[5] CIBER Physiopathology of Obesity and Nutrition (CIBERobn), 14004 Cordoba, Spain
* Correspondence: rgiraldez@uco.es (R.M.G.-P.); elia@us.es (E.G.)
† Rosa M. Giráldez-Pérez and Elia Grueso contributed equally to this paper.

1. Characterization of 16-Ph-16 cationic gemini surfactant

NMR Characterization

For NMR spectroscopic measurements, the synthesized surfactant was dissolved in 99.95% CDCl3 (~10 mg in 0.7 mL) and transferred into 5 mm NMR sample tubes (Promochem, Wesel, Germany). Spectra were measured on a Bruker DRX- 400 AVANCE spectrometer at 400.13 MHz (1H) or 100.62 MHz (13C) using the Topspin 1.3 (Bruker, Rheinstetten, Germany). For 1D spectra 32k data points were recorded and Fourier transformed to spectra with a range of 15 ppm (1H) and 240 ppm (13C). Two-dimensional COSY, TOCSY, NOESY, HMQC and HMBC spectra were measured with 128 experimental runs, each having 1024 data points each. Appropriate linear forward prediction, sinusoidal multiplication and Fourier transformation led to 2D-spectra with ranges of 12 ppm and 220 ppm for 1H and 13C, respectively. Residual CHCl3 was used as internal standard for 1H (δH 7.24) and CDCl3 for 13C (δC 77.0) spectra. Measurement temperature was 298.1 K +/-0.1 K.

N,N′-Di-n-hexadecyl-N,N,N′,N′-tetramethyl-phenylene-1,4-dimethylenammonium dibromide (p-16-Ph-16). 1H-NMR (400 MHz, CDCl3, δ in ppm): 7.79 (s, br, 4 H, H-2 (4x)); 5.10 (s,br, 4 H, H-1′ (2x)); 3.51 (s,br, 4 H, H-1′′ (2x)); 3.21 (s,br, 12 H, CH3 (4x)); 1.80 (s,br, 4 H, H-2′′ (2x)); 1.35 (s,br, 4 H, H-3′′ (2x)); 1.24c (m,br, 48 H, H-4′′ to H-15′′ (2x)); 0.68 (t, 6 H, J=7.0 Hz, H-16′′ (2x)). 13C NMR (100 MHz, CDCl3, δ in ppm): 133.6 (d, C-2 (4x)); 128.7 (s, C-1 (2x)); 66.6 (t, C-1′ (2x)); 65.1 (t, C-1′′ (2x)); 49.6 (q, CH3 (4x)); 31.4 (t, C-14′′ (2x)); 29.7-29.4 (t (10x), C-4′′ to C-13′′ (2x)); 28.5 (t, C-3′′ (2x)); 26.4 (t, C-2′′ (2x)); 22.7 (t, C-15′′ (2x)); 14.1 (q, C-16′′ (2x)).

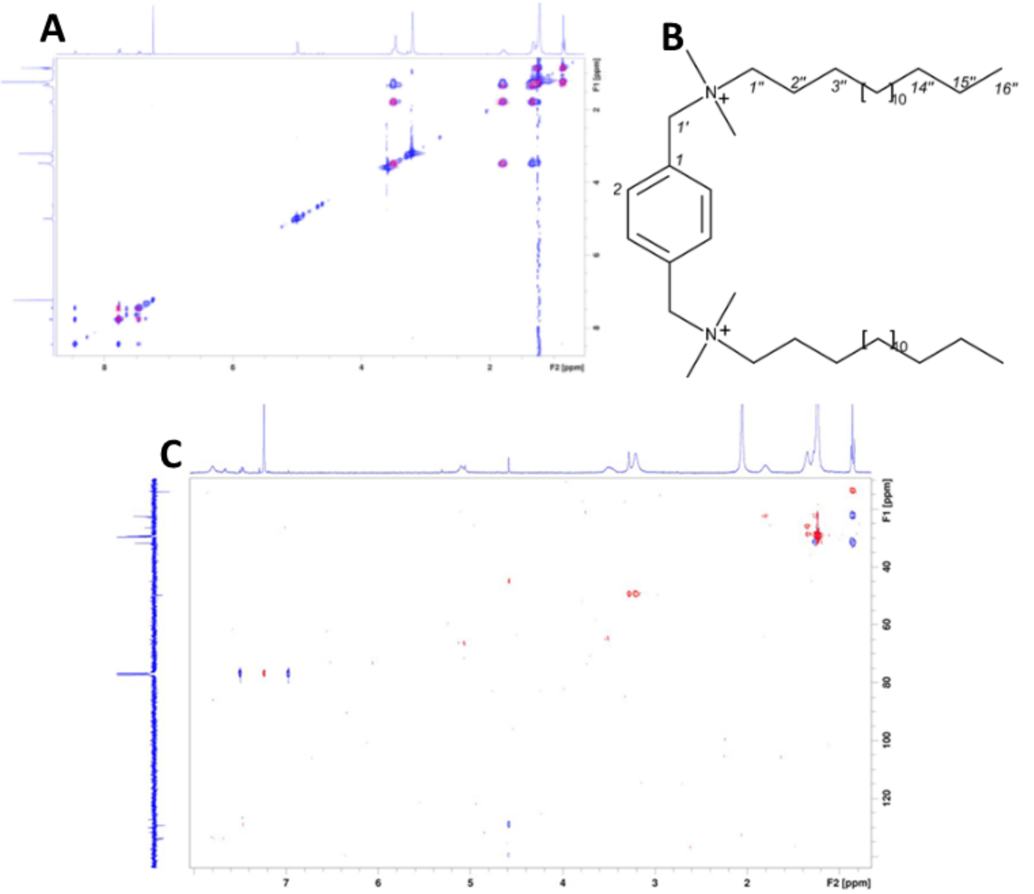

Figure S1. (A) Combined COSY (pink) and TOCSY (blue) spectra of compound 16-Ph-16 together with the respective 1H NMR spectrum on both axes. (B) Structure of the compound 16-Ph-16. The numbering is in accordance with that used for the NMR shift data. It is not in accordance with IUPAC nomenclature but allows an easy comparison of different shifts in the compound. (C) Combined HSQC (red) and HMBC (blue) spectra of compound 16-Ph-16, together with the respective 1H and 13C NMR spectra on the axes

2. TEM Protocol

First, the samples were immersed in a sodium cacodylate trihydrate solution 0.1 M and pH = 7.4 for 5 min at 277.0 K for three times (X3). Samples were then post-fixed with a 1% osmium tetroxide solution (18453, Ted Pella, Inc., Redding, CA, USA) for 1 h at 277.0 K to preserve cell structure, and then they were washed in distilled water for 20 min at 277.0 K for three times (X3). Processing continued with contratinction with a 2% uranyl acetate solution to contrast the sample (22400, Electron Microscopy Sciences, Hatfield, PA, USA) for 2 h at 277.0 K. Next, the samples were dehydrated for subsequent inclusion in resins by washing in distilled water and acetone solution (10000, Electron Microscopy Sciences, Hatfield, PA, USA), increasing gradation from 50% to 90% for 30 min at 298.0 K and two immersions in 100% acetone for 20 min at 298.0 K. Inclusion began with immersion in a mixture of acetone and Spurr resin (14300, Electron Microscopy Sciences, Hatfield, PA, USA) at a 3:1 ratio for 1 h, followed by a mixture of acetone and Spurr resin at a 1:1 ratio for 2 h and another immersion in the same mixture at a 1:3 ratio for 2 h. The samples were then given three subsequent immersions in 100% Spurr resin lasting 2, 12, and 1 h, respectively, the inclusion being processed at 298.0 K for each step. Finally, they remained at 343.0 K for 7 h for the polymerization of the resins. Next, we proceeded to the recutting and semi-fine cuts with a glass blade (UC7 Ultramicrotome, Leica, Nussloch, Germany) in a standard range of 300 nm. To determine areas for ultra-fine cuts, we proceeded to mount sections on standard glass slides and used monochromatic staining with toluidine blue

(251176.1606, Panreac Química S.L.U, Barcelona, Spain). Then ultra-fine cuts were made with a diamond blade that is less than or equal to 70 nm.

3. Confocal Microscope Protocol

With two confocal channels for fluorescence, the microscope was equipped with highly sensitive detectors (QE (quantum efficiency) 70% or better) and a bright-field transmitted-light mode capability. There were individually variable confocal pinholes for each detection channel, and all lasers were of the maintenance-free diode or solid-state type without significant heat dissipation: a 405 nm laser diode at 50 mW, a 488 nm laser diode at 100 mW, a 532 nm diode pumped solid state laser at 75 mW, a 561 nm laser diode at 40 mW, and a 635 nm laser diode at 30 mW. Additional outlet from an existing LIVE Laser Module with polarization preserving single mode fiber; the splitting proportion between the outlets was freely variable via the software for the 405, 488, 532, and 561 nm laser lines.

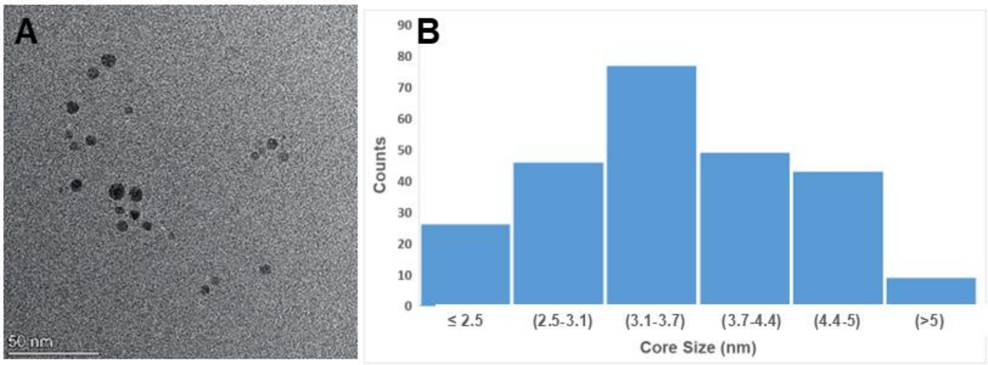

Figure S2. (A) TEM image of free Au@16-Ph-16 gold nanoparticles. (B) Size distribution of Au@16-Ph-16 in water.

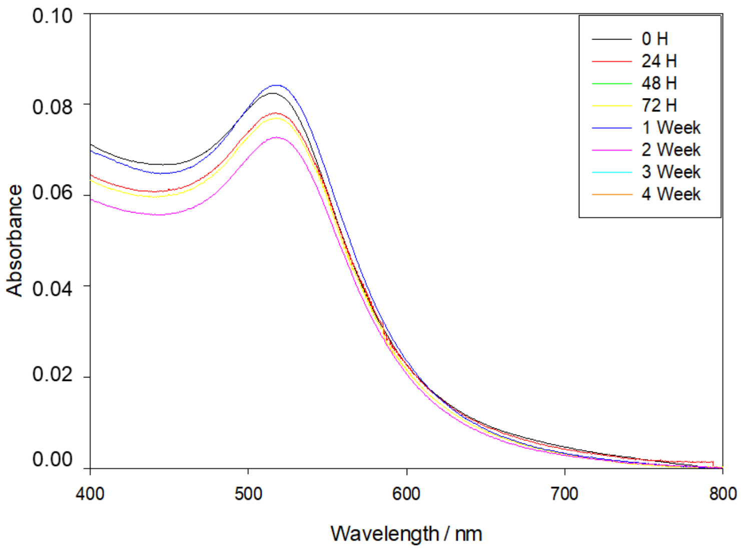

Figure S3. Stability of Au@16-pH-16 from its synthesis to one month.

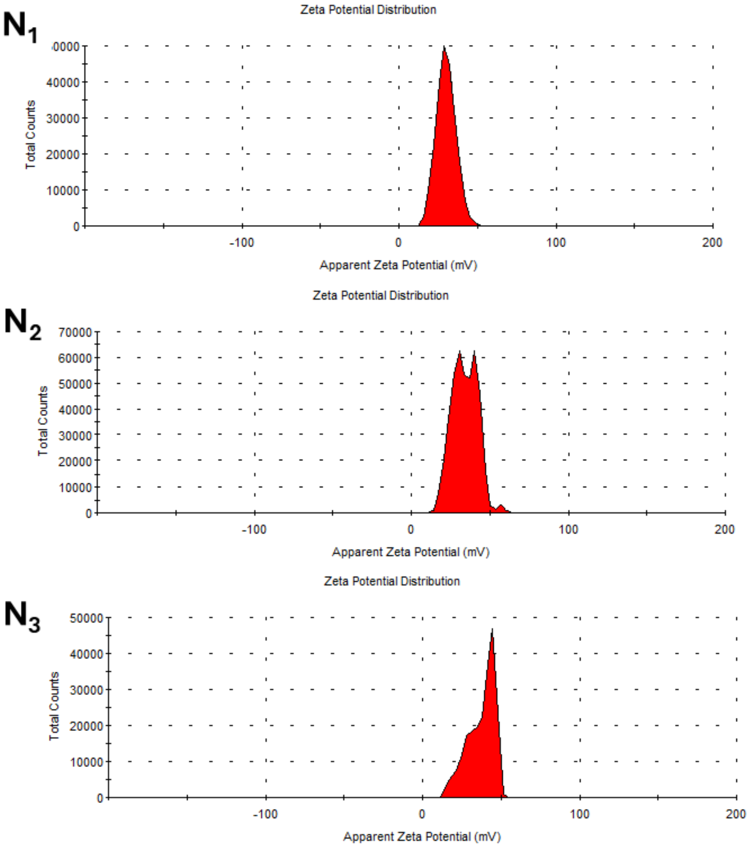

Figure S4. Zeta potential of Au@16-Ph-16 nanoparticles at different Au@16-Ph-16 concentrations. (A) N_1, (B) N_2 and (C) N_3.

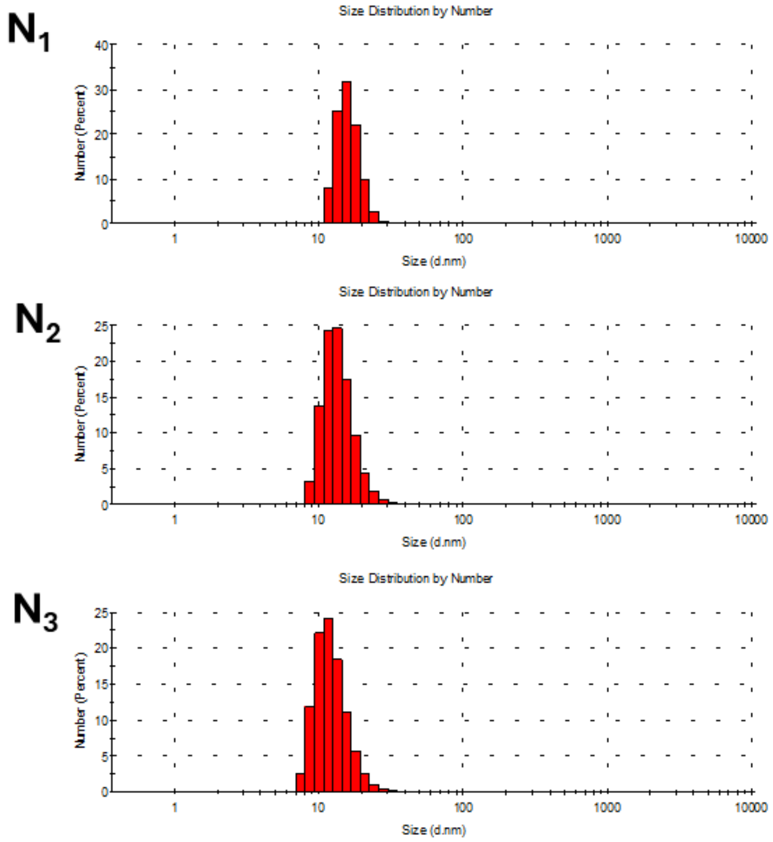

Figure S5. DLS size distribution by number of Au@16-Ph-16 for different formulations. (A) N_1, (B) N_2 and (C) N_3.

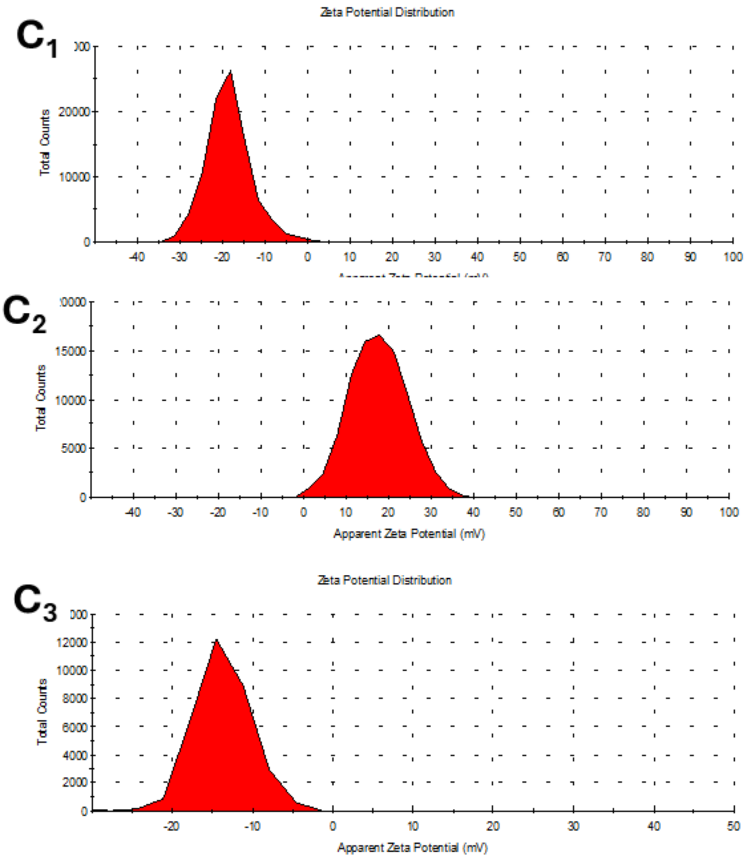

Figure S6. Zeta potential of Au@16-Ph-16/DNA-Dauno compacted nanocomplexes for different formulations. (A) C_1, (B) C_2 and (C) C_3.

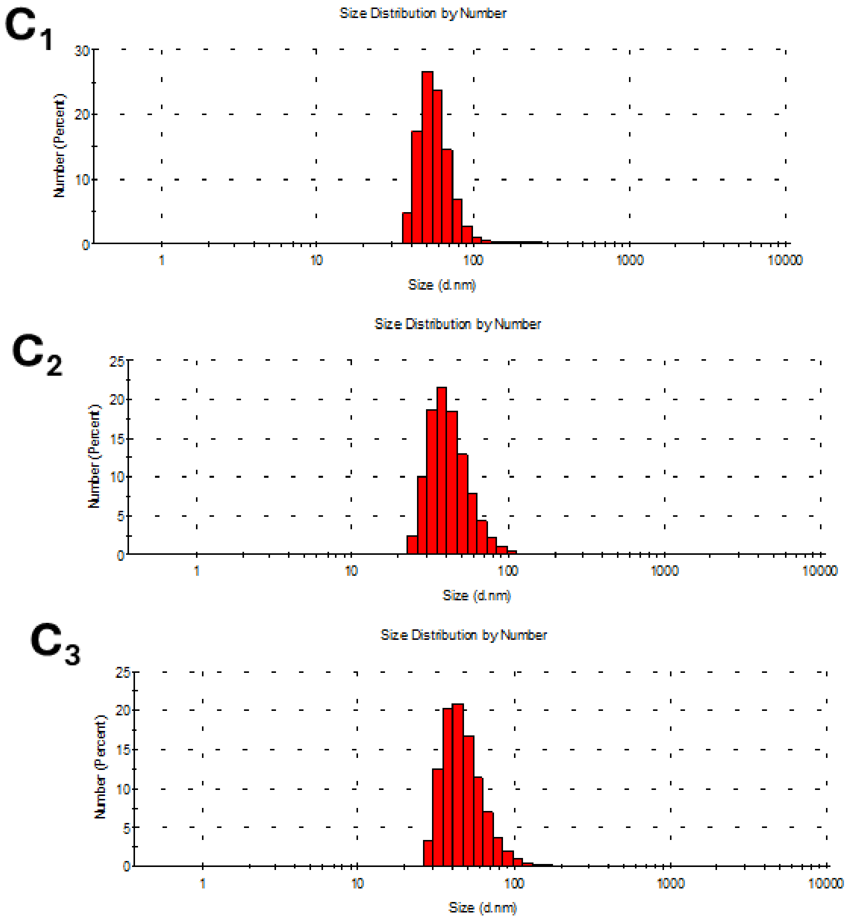

Figure S7. DLS size distribution by number of Au@16-Ph-16/DNA-Dauno compacted nanocomplexes for different formulations. (A) C_1, (B) C_2 and (C) C_3.

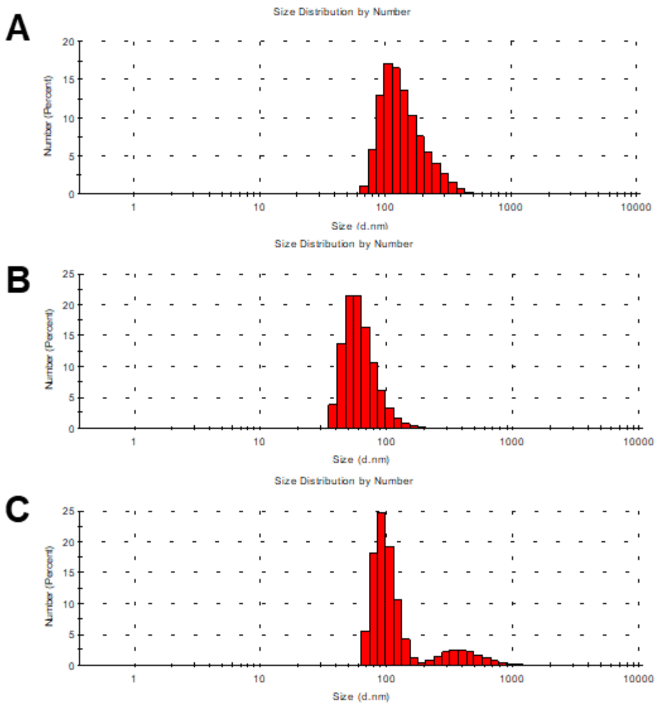

Figure S8. Au@16-Ph-16 dependence on the hydrodynamic diameter of Au@16-Ph-16/DNA–Dauno nanocomplexes at T = 25.0 °C (C_{DNA} = 0.3 μM, C_{Dauno} = 0.03 μM). (A) $C_{Au@16\text{-}Ph\text{-}16}$ = 0.00066 nM. (B) $C_{Au@16\text{-}Ph\text{-}16}$ = 0.0066 nM. (C) $C_{Au@16\text{-}Ph\text{-}16}$ = 0.0660 nM.

Table S1. Diluted concentrations used for both viability and internalization studies.

Compound	Concentration	Compound	Concentration
**N_1'**	$1.19 \cdot 10^{-10}$ M	**C_1'**	[Dauno]= $2.5 \cdot 10^{-8}$M<br>[DNA] = $2.5 \cdot 10^{-7}$ M<br>[Au@16-Ph-16] = 5.5·10-12 M
**N_2'**	$4.78 \cdot 10^{-10}$ M	**C_2'**	[Dauno] = $1.0 \cdot 10^{-7}$M<br>[DNA] = $1.0 \cdot 10^{-6}$ M<br>[Au@16-Ph-16] = $2.2 \cdot 10^{-11}$ M
**N_3'**	$5.6 \cdot 10^{-10}$ M	**C_3'**	[Dauno] = $1.5 \cdot 10^{-7}$M<br>[DNA] = $1.5 \cdot 10^{-6}$ M<br>[Au@16-Ph-16] = $3.3 \cdot 10^{-11}$ M

Table S2: Zeta potential and PDI values of the precursor Au@16-Ph-16 nanoparticles and distinct Dauno nanocarriers, Ci formulation in serum and PBS buffer (0.01×, ionic strength = 1.63 mM, and pH = 7.4) media.

Au@16-Ph-16 precursor	Zeta potential (mV)	PDI
N_1	-5.9 ± 1.2	0.47
N_2	-3.3 ± 0.4	0.48
N_3	20.7 ± 1.8	0.42
**Au@16-Ph-16/DNA-Dauno**	**Zeta potential (mV)**	**PDI**
C_1	-11.6 ± 0.7	0.26
C_2	-12.6 ± 1.1	0.28
C_3	-7.4 ± 1.2	0.42

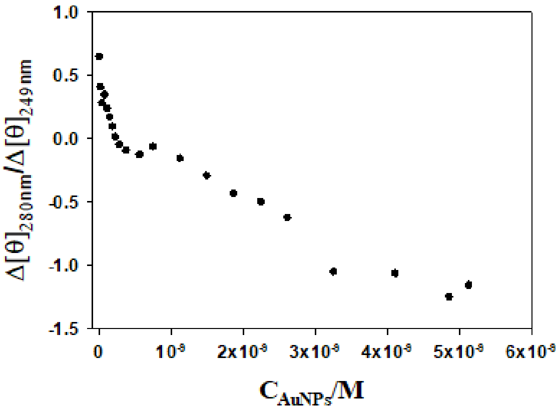

Figure S9. Changes in molar ellipticity ratio between 280 nm and 249 nm of DNA versus the gold nanoparticle concentration.

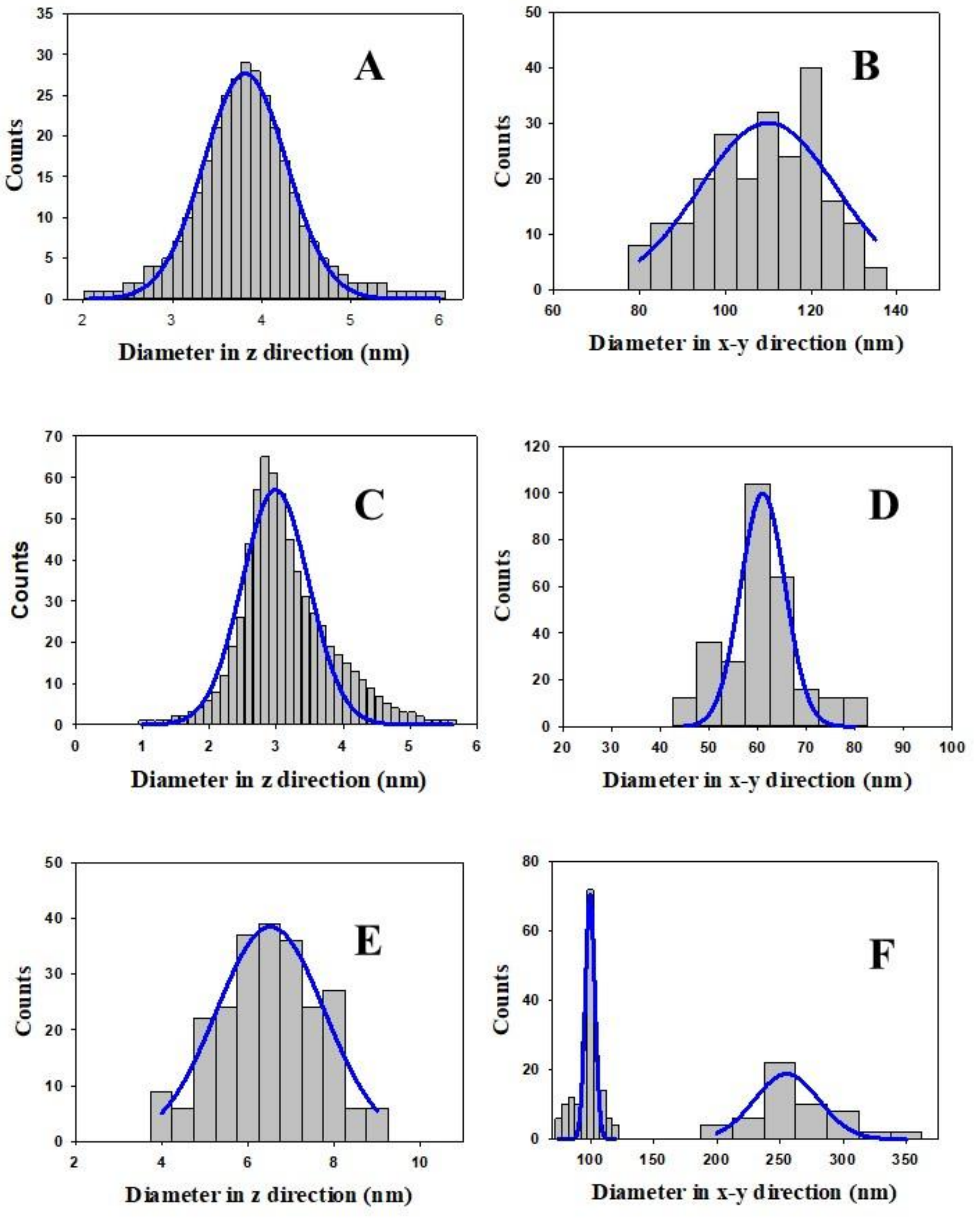

Figure S10. Height/length histograms in z and X-Y direction for AFM topographic samples in Figure 4 of the manuscript, at least 200 particles were measured for each diagram (C_{DNA} = 0.3 µM, C_{Dauno} = 0.03 µM in all the samples). (A-B) $C_{Au@16\text{-}Ph\text{-}16}$ = 0.00066 nM; (A-B) $C_{Au@16\text{-}Ph\text{-}16}$ = 0.0066 nM, and (A-B) $C_{Au@16\text{-}Ph\text{-}16}$ = 0.066 nM.
